# TET2 promotes tumor antigen presentation and T cell IFN-**γ**, which is enhanced by vitamin C

**DOI:** 10.1172/jci.insight.175098

**Published:** 2024-11-22

**Authors:** Meng Cheng, Angel Ka Yan Chu, Zhijun Li, Shiyue Yang, Matthew D. Smith, Qi Zhang, Nicholas G. Brown, William F. Marzluff, Nabeel Bardeesy, J. Justin Milner, Joshua D. Welch, Yue Xiong, Albert S. Baldwin

**Affiliations:** 1 Curriculum in Genetics and Molecular Biology, and; 2 UNC Lineberger Comprehensive Cancer Center, University of North Carolina at Chapel Hill, Chapel Hill, North Carolina, USA.; 3 Department of Computational Medicine and Bioinformatics, University of Michigan, Ann Arbor, Michigan, USA.; 4 Department of Biochemistry and Biophysics, and; 5 Department of Pharmacology, University of North Carolina at Chapel Hill, Chapel Hill, North Carolina, USA.; 6 Department of Medicine, Massachusetts General Hospital, Harvard Medical School, Boston, Massachusetts, USA.; 7 Department of Microbiology and Immunology, University of North Carolina at Chapel Hill, Chapel Hill, North Carolina, USA.; 8 Department of Computer Science and Engineering, University of Michigan, Ann Arbor, Michigan, USA.; 9 Cullgen, Inc., San Diego, California, USA.; 10 Department of Biology, University of North Carolina at Chapel Hill, Chapel Hill, North Carolina, USA.

**Keywords:** Immunology, Oncology, Adaptive immunity, Antigen presentation, Cancer

## Abstract

Immune evasion by tumors is promoted by low T cell infiltration, ineffective T cell activity directed against the tumor, and reduced tumor antigen presentation. The TET2 DNA dioxygenase gene is frequently mutated in hematopoietic malignancies and loss of TET enzymatic activity is found in a variety of solid tumors. We showed previously that vitamin C (VC), a cofactor of TET2, enhances tumor-associated T cell recruitment and checkpoint inhibitor therapy responses in a TET2-dependent manner. Using single-cell RNA sequencing (scRNA-seq) analysis performed on B16-OVA melanoma tumors, we have shown here that an additional function for TET2 in tumors is to promote expression of certain antigen presentation machinery genes, which is potently enhanced by VC. Consistently, VC promoted antigen presentation in cell-based and tumor assays in a TET2-dependent manner. Quantifying intercellular signaling from the scRNA-seq dataset showed that T cell–derived IFN-γ–induced signaling within the tumor and tumor microenvironment requires tumor-associated TET2 expression, which is enhanced by VC treatment. Analysis of patient tumor samples indicated that TET activity directly correlates with antigen presentation gene expression and with patient outcomes. Our results demonstrate the importance of tumor-associated TET2 activity as a critical mediator of tumor immunity, which is augmented by high-dose VC therapy.

## Abstract 2

Tumor-associated TET2 activity drives antigen presentation and promotes tumor-associated T cell activity, which is enhanced by vitamin C therapy. Loss of TET2 in human tumors leads to loss of antigen presentation gene expression and to poor outcomes.

## Graphical abstract

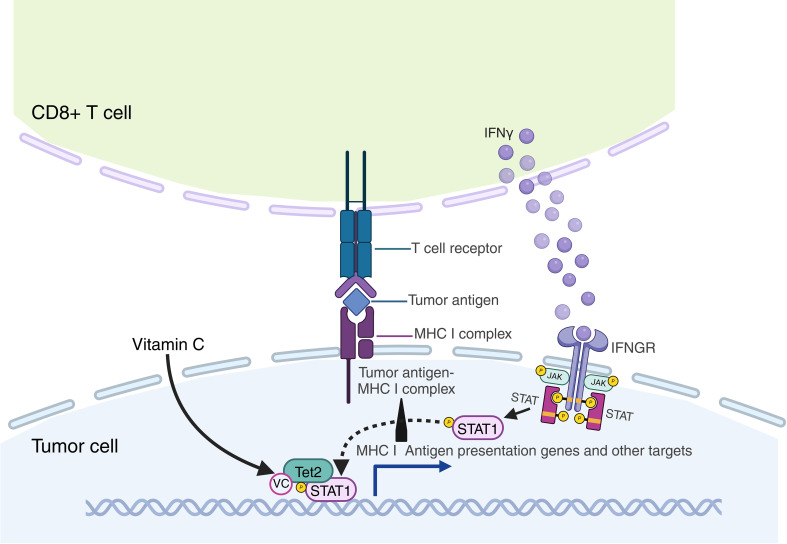

## Introduction

CD8^+^ T cells are the primary immune cells that target and eradicate tumors. Upon recognition of cognate antigen, CD8^+^ T cells give rise to effector cells that migrate to tumor sites to kill cancer cells (1). The recognition of tumor cells by the T cell receptor (TCR) relies on the antigen presentation process on tumor cells via major histocompatibility complex (MHC) class I molecules (1). Antigen presentation by MHC class I molecules involves the degradation of endogenous proteins by the immunoproteasome, which is similar to the 20S proteasome while containing specific subunits, including PSMB8, PSMB9, and PSMB10 that digest proteins into peptides (2). These peptides are translocated through the transporters associated with antigen processing (TAPs) into the endoplasmic reticulum (ER), where they are further trimmed by ER aminopeptidase and loaded onto MHC complexes facilitated by the peptide loading complex (PLC) consisting of TAP and ER chaperones. Tapasin (TAPBP) functions to form stable binding between the peptides and the groove of HLA-I heavy chain α1 and α2 domains on the MHC I complex. The binding of a high-affinity peptide results in the dissociation of the HLA complex from the PLC and its subsequent translocation to cell membranes (3).

Many solid tumors exhibit reduced tumor-directed T cell activity, resulting in immune evasion and ineffective immunotherapy responses (4–7). For example, some tumors overexpress inhibitory ligands such as PD-L1 to suppress T cell activity through its receptor PD-1, forming the basis for PD-1/PD-L1 immune checkpoint blockade as an immunotherapy (1, 4). As a distinct mechanism, tumor cells may downregulate the MHC class I antigen presentation pathway to escape T cell recognition and subsequent killing (4, 5, 8). Mutations in antigen-processing pathway genes or in genes controlling the IFN-γ pathway have been identified in cancers (9) and repression of class I MHC genes through the EZH2-containing PRC2 complex has been reported (10). Importantly, lack of expression of components of the antigen presentation pathway correlates with poor patient outcomes (11–13). While mutations in genes encoding antigen presentation machinery are found in tumors (3), repression of expression of these genes through epigenetic mechanisms is more common (14, 15).

The lack of T cell infiltration into tumors and associated lack of T cell activity are barriers to tumor immunity and responses to immunotherapies (1, 4). As an example, biopsies of melanoma prior to therapy showed that the presence of tumor-infiltrating lymphocytes directly correlates with immune checkpoint inhibitor responses (16, 17). Additionally, expression of T cell–derived IFN-γ predicts response to checkpoint inhibitors (8, 18). Several approaches have been reported to enhance tumor-associated T cell recruitment and frequency of antigen-specific T cells, including the use of oncolytic viruses, immune adjuvants, STING agonists, and tumor-directed vaccines (4).

There are 3 ten-eleven translocation (TET) family proteins, namely TET1, TET2, and TET3, and numerous histone demethylases in mammalian cells, which all play critical roles in regulating gene expression (19–21). The TET proteins function as active DNA demethylases to iteratively convert 5-methylcytosine (5mC) to 5-hydroxylmethylcytosine (5hmc), then 5-formalcytosine (5fC), and finally 5-carboxylcytosine (5caC) (22, 23). TET proteins are recruited to promoters to demethylate DNA through interactions with sequence-specific transcription factors to promote gene expression (24, 25). *TET2* is frequently mutated in lymphoid and myeloid malignancies, leading to loss-of-function phenotypes (26–28). In solid tumors, TET2 loss-of-function mutations are not commonly observed; however, TET enzymatic activity is often low, consistent with an overall low level of genomic 5hmC in some cancers (29–31). One mechanism for reduced activity of TET2 in tumors is through mutations in isocitrate dehydrogenase 1 (IDH1) or IDH2, which lead to production of 2-hydroxyglutarate instead of α-ketoglutarate (α-KG), a required TET2 cofactor (32). Vitamin C (VC) is known as an essential dietary nutrient with well-established immunomodulatory and antioncogenic effects (19, 33). VC acts as a cofactor for the TET family of DNA dioxygenases and Jmjc family of histone demethylases that are involved in active DNA or histone demethylation, respectively (34, 35). We and others have shown that VC binds directly to TET2 and promotes its catalytic activity (36, 37).

We previously reported the effect of VC in promoting checkpoint inhibitor immunotherapy efficacy using the B16-OVA melanoma syngeneic tumor model and that this is dependent on tumor-associated TET2 expression (38). Furthermore, we showed that VC increases chemokine gene CXCL9/-10/-11 expression in a TET2-dependent manner, leading to type I T cell attraction to tumor sites and to enhanced checkpoint inhibitor responses (38). Others subsequently showed that VC enhances tumor immunotherapy responses (39, 40). Additionally, Bardeesy and colleagues (41) showed that restoration of TET activity in an IDH-mutant cholangiocarcinoma tumor model restored downstream IFN-γ signaling and enhanced checkpoint inhibitor responses.

Using single-cell RNA sequencing (scRNA-seq) analysis of the B16-OVA melanoma model, we have shown here that TET2 has an additional function in tumors, namely driving the expression of genes encoding proteins involved in antigen processing and presentation. VC, dependent on TET2, promoted expression of these genes, correspondingly enhancing checkpoint inhibitor therapeutic effects. Consistently, presentation of the OVA tumor antigen was enhanced by TET2 following IFN-γ stimulation, which was further stimulated by VC treatment, and effective in vitro cytotoxic activity of T cells requires TET2 tumor expression. Intercellular communication analysis of the scRNA-seq data showed that TET2 tumor expression was required for T cell activation and subsequent IFN-γ–controlled signaling events, which were strongly enhanced by VC treatment. Consistent with this, IFN-γ treatment of tumor cells promoted TET2 binding to the promoters of antigen presentation genes. Additionally, results indicated a role for TET3 in controlling the activation of some antigen presentation genes. Importantly, analysis of human tumor tissue revealed that loss of TET activity correlates with reduced antigen presentation gene expression and with poor patient outcomes.

## Results

### Intravenous delivery is optimal for VC promotion of immune checkpoint inhibitor therapy.

**Figure 1:**
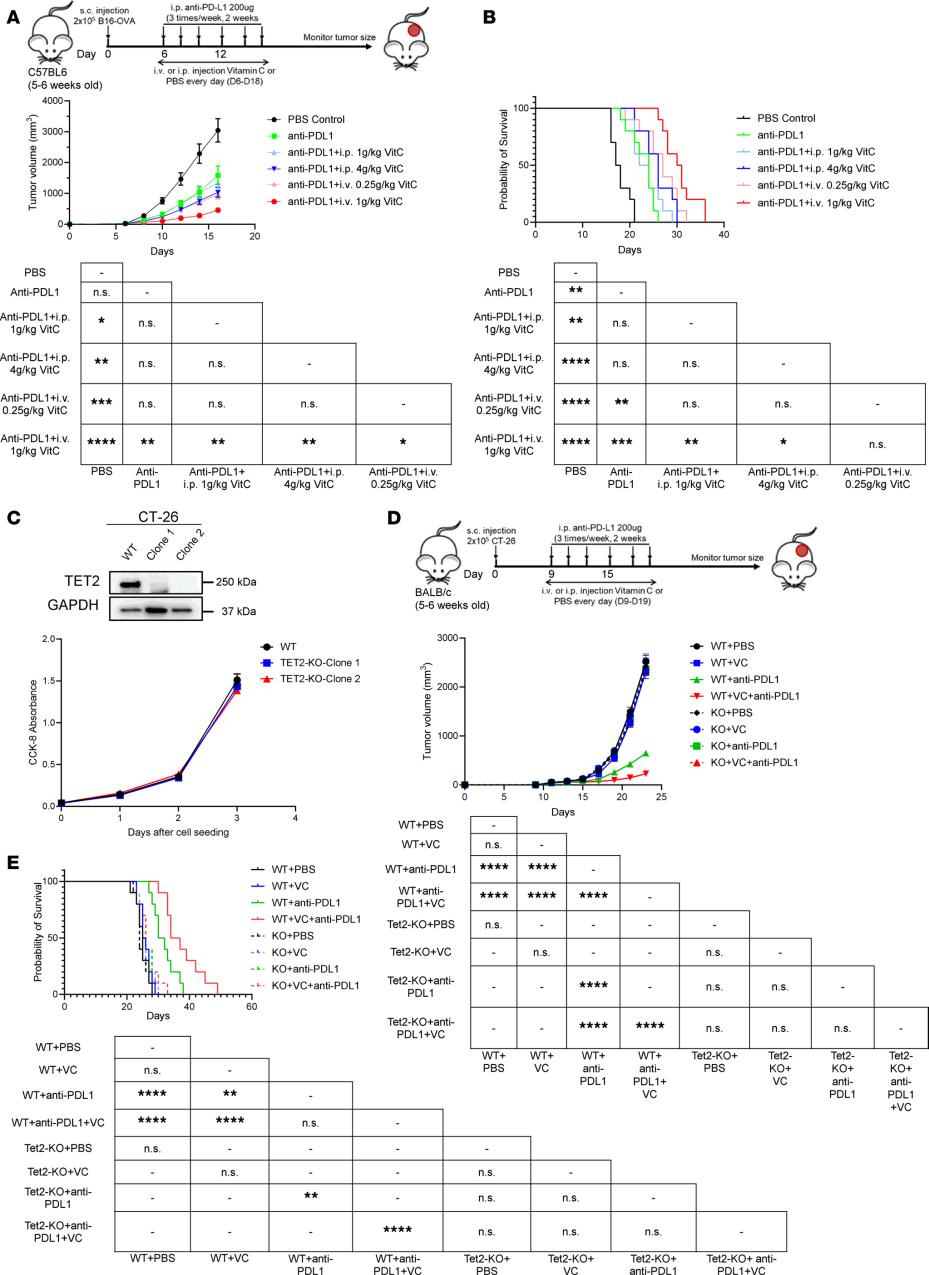
Injection of VC i.v. provides optimal, TET2-dependent anti–PD-L1 immunotherapy efficacy. WT B16-OVA (2 × 10^5^) cells were transplanted into 6-week-old C57BL/6J syngeneic mice followed by indicated doses of VC treatment daily or 200 μg anti–PD-L1 3 times per week. Tumor volume (**A**) was measured (width^2^ × length/2) with a caliper and mouse survival (**B**) was monitored and data recorded every day. Error bars represent ± SEM, *n* = 10. (**C**) TET2-KO clones of CT-26 cells were made using the CRISPR/Cas9 system and were confirmed by Western blotting as well as DNA sequencing; TET2 expression in CT-26 cells did not affect its proliferation in culture medium, *n* = 5. CT-26 (2 × 10^5^) cells were transplanted into 6-week-old BALB/c syngeneic mice followed by 1 g/kg i.v. VC treatment every day or 200 μg anti–PD-L1 3 times per week as indicated. Tumor volume (**D**) was measured with a caliper and mouse survival (**E**) was monitored and data recorded each day. The *P* values shown in the tables for survival data were determined by log-rank (Mantel-Cox) test comparing each 2 groups or unpaired, 2-tailed Student’s *t* test, and multiple comparisons were corrected using Bonferroni’s method. Error bars represent ± SEM, *n* = 10. Statistical significance was defined as an adjusted *P* value (Bonferroni’s correction) of less than 0.05. *P < 0.05, **P < 0.01, ***P < 0.001, ****P < 0.0001. NS, no significance.

High-dose VC treatment has been shown to reduce tumor growth in both human patients and in mouse models (42). We and others reported that intraperitoneal (i.p.) VC administration at 4 g/kg increased immunotherapy efficacy when combined with anti–PD-1/anti–PD-L1 immune checkpoint inhibitor therapies (38–40). In a previous liquid chromatography–mass spectrometry (LC-MS) study (43), 3 different delivery methods for VC were used with C57BL/6J mice, namely oral intake, intraperitoneal (i.p.) injection, and intravenous (i.v.) injection at 1 g/kg. Among these methods, oral intake reached 40 mM for the peak plasma concentration, while both i.p. injection and i.v. injection resulted in greater than 5 mM concentration, with the highest plasma concentration at 20 mM for i.v. injection. Based on this, we compared i.p. VC delivery with i.v. delivery related to effects on tumor growth with or without anti–PD-L1 treatment. Compared with PBS control, anti–PD-L1 treatment alone reduced tumor growth in the B16-OVA syngeneic tumor model, as previously shown (38). When combined with anti–PD-L1 immune checkpoint inhibitor, i.p. injection of VC increased the antitumor effects at 4 g/kg, while a dose of 0.25 g/kg for i.v. injection was sufficient to reach the equivalent response. Moreover, when anti–PD-L1 was administered with 1 g/kg VC through i.v. injection, tumor growth was largely suppressed, and the average tumor volume was less than one-third compared with anti–PD-L1 treatment alone (Figure 1A). Consistent with the tumor growth data, Kaplan-Meier survival analysis indicates that i.v. injection of VC at 1 g/kg produced the optimal survival outcome when combined with PD-L1 checkpoint blockade, while i.p. injection of the same dose did not further enhance mouse survival (Figure 1B). Dependence on TET2 for the effect of VC was shown in our previous study (38). Of note, i.v. injection at a dose of 2 g/kg or higher is toxic. The results demonstrate that i.v. injection of VC is more effective than i.p. injection in enhancing immunotherapy efficacy in the B16 melanoma tumor model.

### Enhanced immunotherapeutic efficacy induced by VC is largely TET2 dependent.

To test the effects of VC relative to dependence on TET2 in a different model, we used the CT-26 colorectal syngeneic tumor model. TET2-KO clones for the CT-26 colon cancer cells were generated using CRISPR/Cas9 genome editing (Supplemental Figure 1A; supplemental material available online with this article; https://doi.org/10.1172/jci.insight.175098DS1) and confirmed by both Western blotting and DNA sequencing (44). Proliferation of the TET2-KO clones was similar to the WT clone, indicating that loss of TET2 expression in CT-26 tumor cells does not affect tumor cell proliferation or survival (Figure 1C). We then transplanted WT and TET2-KO CT-26 cells into syngeneic BALB/c mice and treated with VC, anti–PD-L1, or both. In the WT groups, PD-L1 blockade reduced tumor growth, which was further enhanced by administration of i.v. 1 g/kg VC, while no effect was seen for the combined treatment in the TET2-KO groups (Figure 1D). Tumor growth measurements indicate that neither VC nor anti–PD-L1 worked to inhibit tumor growth in the TET2-KO groups when compared with the PBS control. Moreover, Kaplan-Meier survival data indicate that anti–PD-L1 immunotherapy enhanced survival in the WT groups, but not in the TET2-KO groups. Consistent with the tumor growth data, VC combined with anti–PD-L1 yielded optimal survival only in the TET2-WT tumors (Figure 1E).

### VC promotes expression of tumor antigen presentation genes in a TET2-dependent manner.

**Figure 2:**
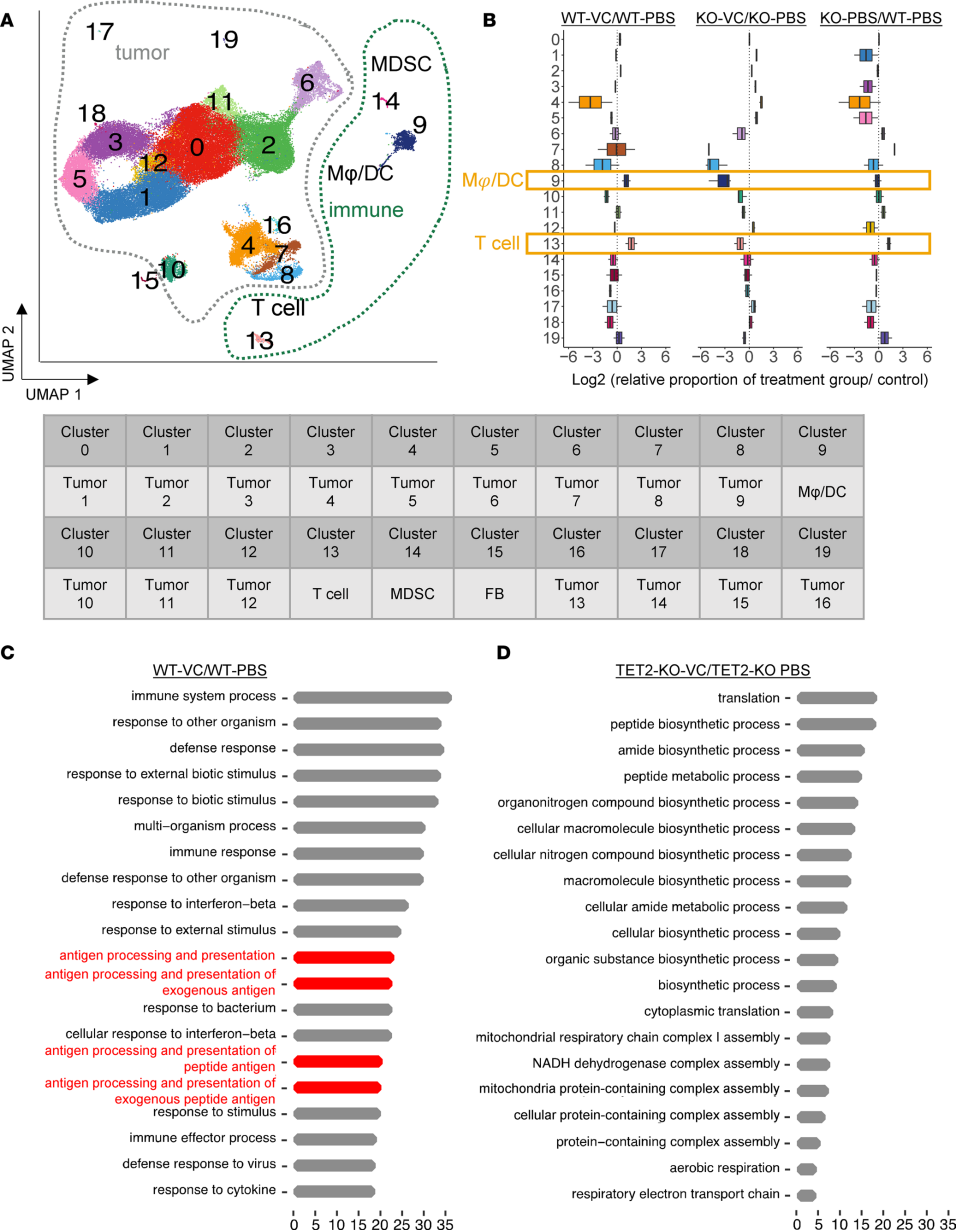
scRNA-seq reveals enhanced antigen presentation processes after VC treatment in WT, but not TET2-KO, B16-OVA syngeneic tumor tissue. (**A**) UMAP clustering from the total population of WT or TET2-KO B16-OVA treated with PBS or i.v. 1 g/kg VC groups (3 replicates per group with approximately 10,000 single cells per sample were sequenced for all 4 groups) was summarized and a total of 20 different clusters were identified, including myeloid-derived suppressor cell (MDSC), dendritic cell (DC), macrophage (Mφ), T cell, and fibroblast (FB) cell defined by their marker gene expression. (**B**) Population changes after VC treatment for each cluster were summarized in the bar plot. The relative abundance of each cluster was calculated by their ratio in that sample divided by the corresponding PBS control group. The top 20 most significantly enriched cellular processes based on GO analysis of the top 100 DEGs after VC treatment in the WT group (**C**) or TET2-KO groups (**D**) in the whole tumor tissue were summarized and antigen presentation processes are marked in red. Horizontal axes in **C** and **D** show the qscore.

To analyze the effects of VC and TET2 on the tumor microenvironment and on gene expression responses in tumor cells, we performed scRNA-seq analysis. We transplanted TET2-WT and TET2-KO B16-OVA tumor cells into C57BL/6J syngeneic mice and treated the mice daily with i.v. 1 g/kg VC or PBS as a control. Delivery of VC via the i.v. route was chosen due to its efficiency over i.p. delivery. Tumors from these 4 groups were collected and subjected to scRNA-seq analysis. We identified a total of 20 different clusters using Uniform Manifold Approximation and Projection (UMAP) clustering based on the marker genes they express (Figure 2A). Among these 20 clusters, 15 are different tumor subtypes, accounting for more than 90% of total sequenced cells. Interestingly, there was a configuration consisting of 4 different tumor subtypes, as shown in the UMAP, namely clusters 1, 3, 5, and 12. Further differentially expressed gene (DEG) analysis for these 4 clusters revealed that they represent different phases of mitosis, suggesting rapid tumor growth in the B16-OVA syngeneic tumor model. In addition to the tumor clusters, we identified several non-tumor clusters: cluster 9, cluster 11, cluster 13, cluster 14, and cluster 15. Cluster 9 consists of macrophages and dendritic cells plus an uncharacterized subset of cells (see Supplemental Figure 2, A and B, and Methods), representing the most abundant immune cell type, while cluster 13 is mainly T cells. The cluster 9 and cluster 13 populations were modestly increased after VC treatment in the WT, but not in the TET2-KO, tumor background when comparing the relative ratio of different immune cell clusters (Figure 2B).

Next, we focused on the genes and pathways that are altered by VC treatment in the WT tumors. As compared with PBS control tumors, VC administration led to contrasting patterns in gene expression. Among the top 20 pathways induced by VC, 4 are involved in antigen presentation processes as identified by Gene Ontology (GO) analysis, suggesting that the antigen presentation process is a major target for VC (Figure 2C). Other pathways induced by VC include innate immune response to IFNs and innate immune regulation, consistent with reports for the importance of TET2 in IFN responses (38). Interestingly, in the TET2-KO groups, antigen presentation and IFN response processes were not found in the top 20 enriched pathways after VC stimulation, but included processes associated with metabolism and biosynthesis (Figure 2D). Together, these data suggest that the ability of VC to enhance T cell responses is partly dependent on TET2-mediated upregulation of antigen presentation genes and machinery in tumor cells.

### Increased antigen presentation pathway gene expression is TET2 dependent in tumor cells.

**Figure 3:**
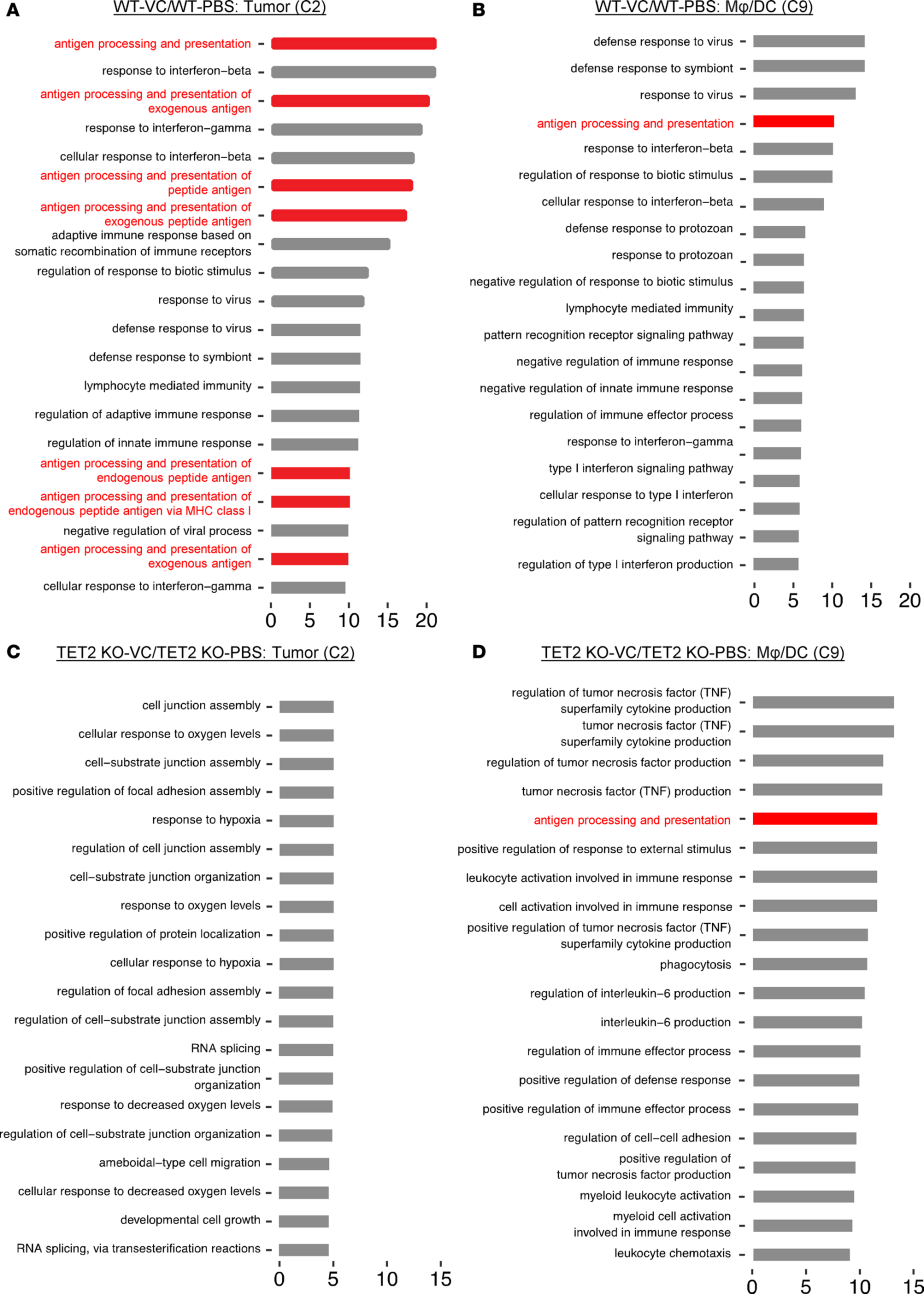
VC-induced antigen presentation processes are largely from tumor cells and rely on the expression of TET2. The top 20 most significantly enriched cellular processes based on GO analysis of the top 100 DEGs after VC treatment in the WT tumor cluster C2 (**A**) or WT group macrophage/dendritic cell cluster C9 (**B**) and in the TET2-KO tumor cluster C2 (**C**) or TET2-KO group macrophage/dendritic cell cluster C9 (**D**) were summarized, and antigen presentation processes are marked in red. Horizontal axes show the qscore.

To determine whether the VC-induced antigen presentation processes occur in tumor cells or in antigen-presenting cells, we performed GO pathway analysis for each scRNA-seq cluster. As shown for tumor cluster 2, antigen presentation–related processes were among the top 20 enriched pathways upregulated by VC (Figure 3A). Most other tumor clusters also displayed a similar result (data not shown). However, for cluster 9, which contains macrophages and dendritic cells as the major source of antigen-presenting cells in the tumor tissue, one antigen presentation–related pathway was shown to be induced by VC treatment among the top 20 (Figure 3B and see Discussion). Additionally, innate immune responses and a variety of external stimuli and their corresponding regulation were the major effects of VC in cluster 9 (Figure 3B). Taken together, these results demonstrate that the increase in expression of antigen presentation pathway genes by VC in the WT tumor tissue is largely associated with tumor cells, but also occurs in some immune cells.

As described above, antigen presentation pathways were largely unaffected in the TET2-KO tumor tissue regardless of VC treatment. To further validate this, we performed GO analysis for each cluster in the TET2-KO tumors. In tumor cluster 2, antigen presentation processes were not identified among the top 20 enriched pathways, supporting the hypothesis that the increased antigen presentation pathways induced by VC in tumor cells was TET2 dependent (Figure 3C). A similar result was observed for other tumor clusters in the TET2-KO groups as well (data not shown). One antigen presentation process was identified in cluster 9 in the TET2-KO tumor treated with VC among those top 20 enriched pathways (Figure 3D), consistent with previous data showing that loss of TET2 expression in the tumor does not block the effect of VC on the dendritic cell/macrophage cluster. Furthermore, we directly compared the WT tumors to TET2-KO tumors treated with PBS control and found there was an elevation in the basal levels of antigen presentation–related processes in the WT tumor clusters in cluster 0 and cluster 3 (Supplemental Figure 2, C and D). This suggests that TET2 functions to control tumor-associated expression of genes involved in antigen processing/presentation in B16-OVA tumor cells, with further enhancement following VC treatment (and see below). We performed molecular function pathway analysis for tumor clusters C0 and C2 and found major mechanisms associated with antigen presentation functions (such as MHC protein complex binding and MHC class II complex binding) enhanced by VC treatment (Supplemental Figure 2, E and F), which was lost in the TET2-KO tumor clusters (Supplemental Figure 2, G and H).

### VC promotes antigen presentation gene expression in a TET2-dependent manner.

**Figure 4:**
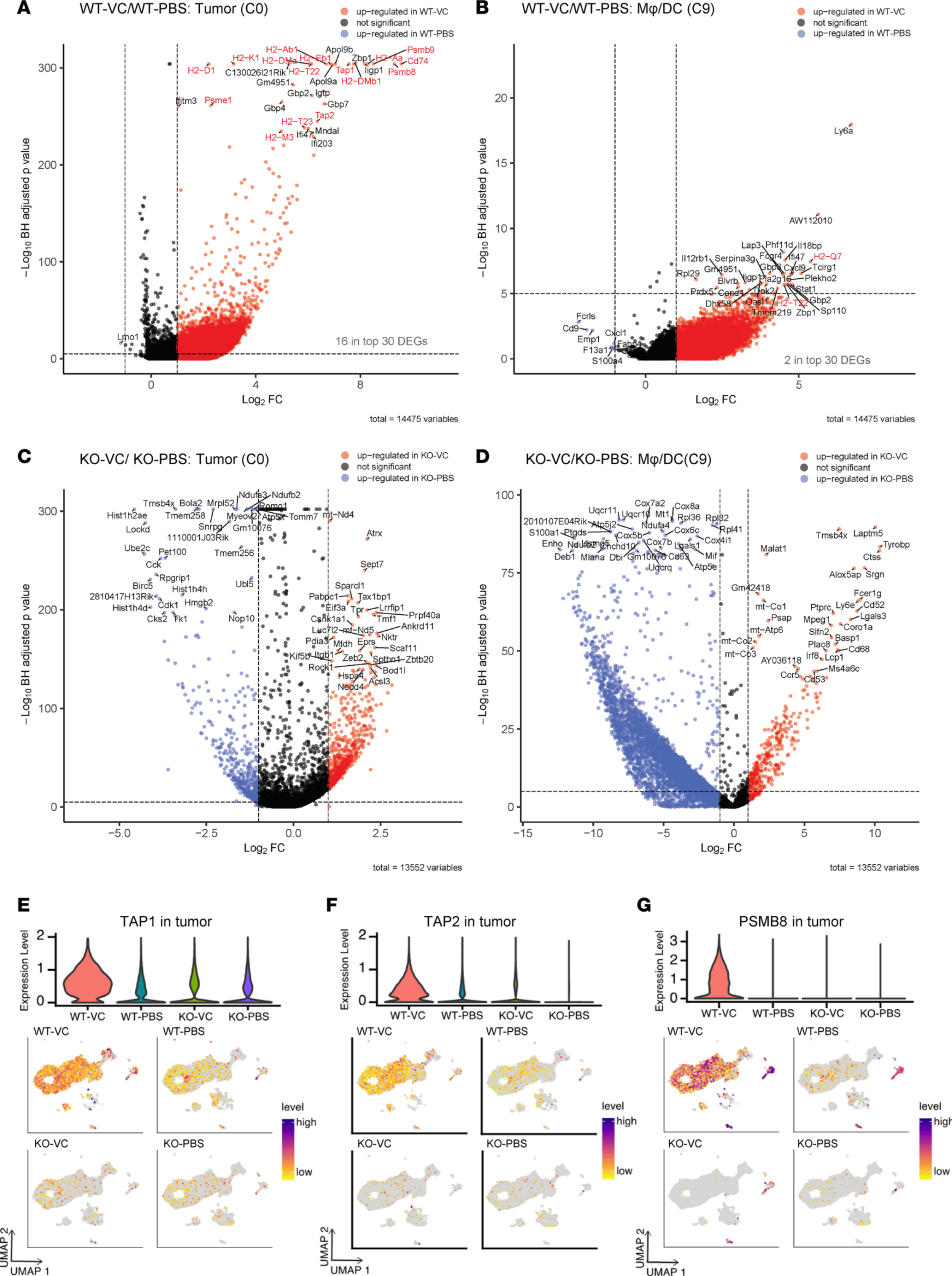
VC induces MHC class I–related antigen presentation gene expression in WT, but not TET2-KO, tumor cells. The top 30 DEGs after VC treatment in WT tumor cluster C0 (**A**) or WT group macrophage/dendritic cell cluster C9 (**B**) and TET2-KO tumor cluster C0 (**C**) or TET2-KO group macrophage/dendritic cell cluster C9 (**D**) were annotated in the volcano plots according to their adjusted *P* values and genes involved in the MHC I antigen presentation are marked in red. The single-cell expression profile of MHC class I genes TAP1 (**E**), TAP2 (**F**), and immunoproteasome gene PSMB8 (**G**) in the WT or TET2-KO B16-OVA tumor tissue treated with PBS control or VC are displayed on the UMAP projection from the whole tumor tissue. The relative expression of genes was calculated by log_2_-normalized gene count data where purple indicates high expression and yellow indicates low expression, with detailed quantification in the violin plots shown above.

To determine whether VC stimulates antigen presentation gene expression in tumor cells, we analyzed the DEGs between VC-treated tumor clusters relative to PBS control in both WT tumors and TET2-KO tumors. Consistent with the pathway analysis, 15 antigen-presenting genes were identified as being stimulated by VC treatment, among those in the top 30 DEGs in tumor cluster 0 as shown in a volcano plot (Figure 4A). These upregulated genes include those encoding multiple H2 class I MHC genes, TAP1 and TAPBP1, immunoproteasome components such as PSMB9, and CD74, which is the class II MHC γ chain involved in antigen presentation. Interestingly, it was reported that class II MHC is active in melanoma and predicts responses to anti–PD-L1 therapy (45). Similarly, antigen presentation genes were also stimulated by VC in other tumor clusters (Supplemental Figure 3, A and B), suggesting a robust induction of antigen-presenting gene expression by VC treatment in the WT tumor cells. However, in non-tumor clusters represented by cluster 9, most of these antigen presentation genes were not among the top 30 DEGs induced after VC treatment (Figure 4B). These data together indicate the increased antigen presentation processes induced by VC treatment are mainly from upregulated antigen-presenting gene expression in the WT tumor cells. Consistent with the previous GO pathway results, VC administration did not induce antigen-presenting gene expression in the TET2-KO groups, either the tumor cells or non-tumor cells (Figure 4, C and D, and Supplemental Figure 3, C and D).

TAP1, TAP2, TAPBP, and β2-microglobulin (B2M) are critical proteins involved in the class I MHC antigen presentation process in virus-infected or tumor cells (46, 47). TAP1 and TAP2 work together at the ER membrane to load peptides degraded by the immunoproteasome onto MHC class I complexes on the ER membrane with help from TAPBP. Then, the mature peptide–bound MHC I complex that contains B2M is assembled and transported to the Golgi body, which is then presented on the cell surface through the membrane transport system (47). Therefore, we also compared the relative expression levels of these key antigen-presenting genes in the scRNA-seq dataset. The expression of TAP1 was shown to be widely expressed in the WT tumor tissue treated with PBS. Compared with PBS control, VC treatment increased the expression of TAP1 in the tumor clusters, but remained largely unchanged between WT and TET2-KO tumors (Figure 4E). TAP1 was not upregulated by VC in the TET2-KO tumor cells. Similarly, the expression of TAP2, TAPBP, PSMB8, and B2M was each shown to be induced by VC treatment in the WT, but not TET2-KO, tumor cells (Figure 4, F and G, and Supplemental Figure 4, A and B). Indeed, numerous MHC class I genes, including H2-D1 (Supplemental Figure 4C), H2-Aa (Supplemental Figure 4D), H2-K1, H2-Ab1, H2-Eb1, H2-M3, H2-DMa, H2-DMb, and H2-T22 (data not shown) were also upregulated by VC in the WT tumor cells. Importantly, expression of these antigen presentation genes was relatively low in TET2-KO tumor cells regardless of VC treatment. Interestingly, the 3 main immunoproteasome genes, PSMB8, PSMB9, and PSMB10, which digest cellular protein to antigenic peptides for antigen presentation (2), were also upregulated by VC in the WT tumor cells, as indicated by PSMB8 (Figure 4G) and others (Supplemental Figure 4, E and F). Consistent with this analysis, these antigen presentation genes were not significantly elevated in WT tumor cells compared with TET2-KO tumors (Supplemental Figure 3E and see Discussion). Together, these results reveal the role of TET2 as a regulator of expression of many antigen presentation and immunoproteasome genes, requiring VC for robust induction.

### VC increases tumor antigen presentation to activate T cell–mediated tumor killing.

**Figure 5:**
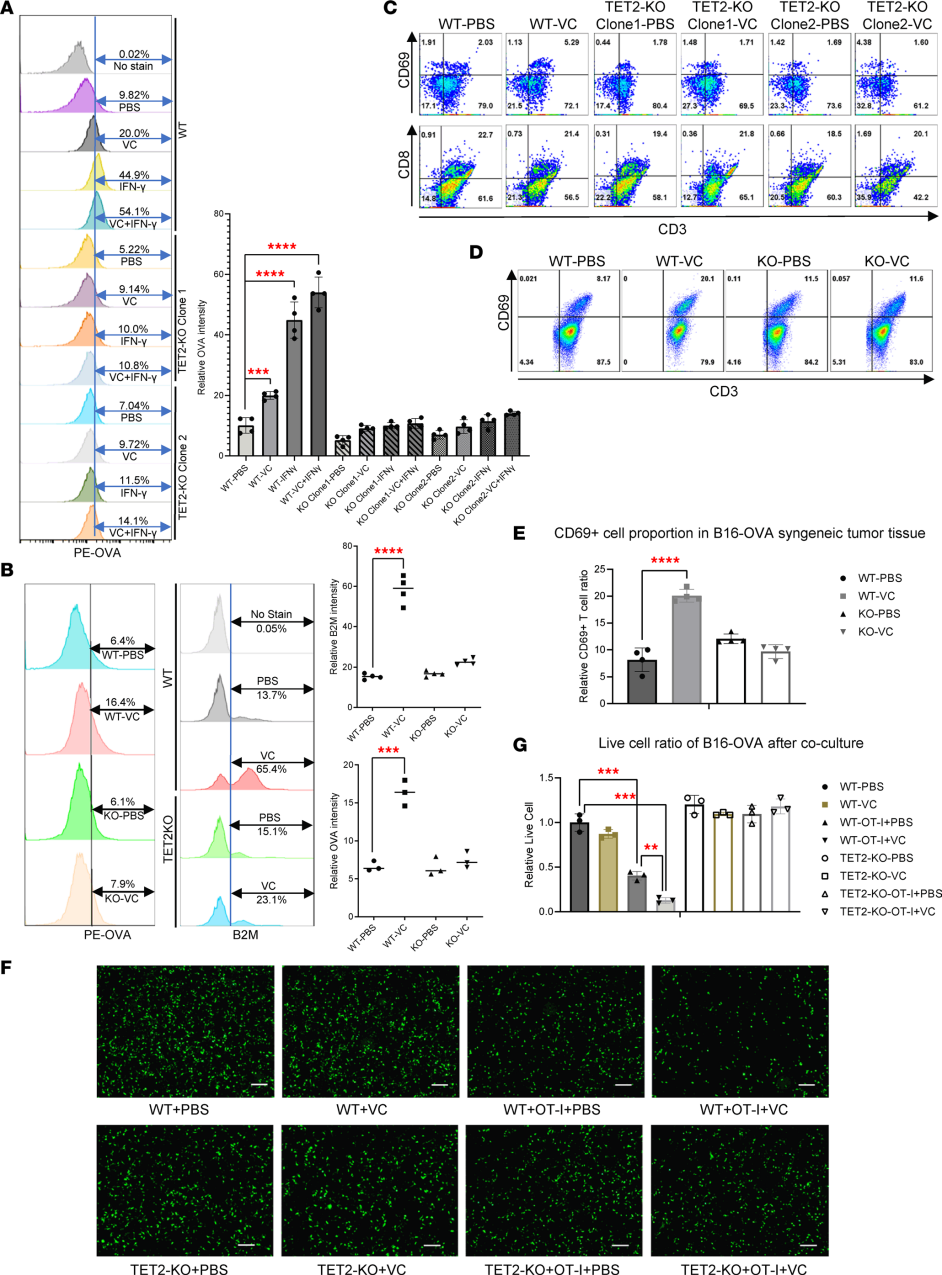
VC increases tumor cell antigen presentation and T cell activation as well as T cell–induced tumor cell killing. (**A**) WT or 2 TET2-KO clones were cultured in 6-well plates in DMEM. Then, 500 μM VC or 100 ng/mL IFN-γ or PBS control were added as indicated for 24 hours before running flow cytometry experiments to determine OVA antigen levels on the tumor cell surface. Data are quantified on the right, with mean ± SD (*n* = 4). *P* values were calculated by unpaired, 2-tailed Student’s *t* test, with multiple comparisons corrected using Bonferroni’s method. (**B**) WT or TET2-KO B16-OVA tumor cells (2 × 10^5^ each) were transplanted into 6-week-old C57BL/6J syngeneic mice and i.v. 1 g/kg VC or PBS treatment was given daily from day 7 to the date of harvesting tumor tissue. Then, single cells were isolated from tumor tissue for flow cytometry experiments to detect OVA antigen presentation or the B2M component of the MHC I complex on the tumor cell surface. Data are quantified on the right, with mean ± SD (*n* = 4). *P* values were calculated by unpaired, 2-tailed Student’s *t* test, with multiple comparisons corrected using Bonferroni’s method. (**C**) WT or TET2-KO B16-OVA cells were cultured in 6-well plates with RPMI medium. Then, 1 × 10^6^ isolated OT-I T cells were cocultured with B16-OVA cells and 500 μM VC or PBS was added as indicated above for the coculture experiments in the presence of 1 × 10^5^ isolated dendritic cells as the antigen-presenting cell. After 16 hours, OT-I T cells were collected for flow cytometry experiments to determine T cell activation using the CD69 marker. (**D**) T cells isolated from WT or TET2-KO B16-OVA syngeneic mice treated with PBS or VC as described in **B** were collected for flow cytometry assays to determine CD69^+^ activated T cell ratio and the results were calculated and summarized (**E**). The error bars indicate 5 replicates in each group and data are represented as mean ± SD. *P* values were calculated by unpaired, 2-tailed Student’s *t* test. (**F**) WT or TET2-KO B16-OVA cells were transfected with EGFP plasmid and then cocultured with isolated OT-I T cells as described in **C**. After 16-hour treatment with PBS or 500 μM VC, cells were washed 3 times with PBS before acquiring images. Scale bars: 100 μm. (**G**) The quantification of live cells from **F** was summarized and data are represented as mean ± SD (*n* = 4). *P* values were calculated by unpaired, 2-tailed Student’s *t* test, with multiple comparisons corrected using Bonferroni’s method. **P < 0.01; ***P < 0.001; ****P < 0.0001.

B16-OVA melanoma cells express the OVA antigen, which is recognized by OT-I (CD8^+^) T cells isolated from OT-I mice (48, 49), making it a relevant model to study tumor-specific CD8^+^ T cell responses. Hence, we treated the B16-OVA melanoma cells with VC and IFN-γ to determine whether VC would enhance OVA antigen presentation in B16-OVA cells. As shown in Figure 5A, VC treatment alone nearly doubled OVA antigen presentation on the surface of WT B16-OVA tumor cells, as detected by flow cytometry. Moreover, when cells were costimulated with IFN-γ, VC was shown to further enhance IFN-γ–induced OVA presentation. Induction of OVA presentation was less robust after VC treatment in 2 TET2-KO B16-OVA clones. In addition, OVA antigen presentation was largely uninduced by IFN-γ in the TET2-KO clones, emphasizing the critical role of TET2 in activating antigen presentation genes in B16-OVA tumor cells. Additionally, loss of TET2 reduced baseline OVA peptide presentation by approximately half (Figure 5A). To test whether VC also induces antigen presentation in tumor tissue, we isolated cells from WT or TET2-KO B16-OVA syngeneic tumor tissue treated with VC or PBS. OVA antigen presentation was increased by more than 3-fold in the WT tumor cells after VC treatment, while remaining largely unchanged in the TET2-KO tumor cells (Figure 5B). We observed increased expression of B2M in the WT tumor cells treated with VC, which was reduced in the TET2-KO cells (Figure 5B). The baseline expression of B2M was not changed between WT and TET2-KO cells (Figure 5B and see below).

To investigate whether increased antigen presentation by VC correlates with enhanced tumor killing by CD8^+^ T cells, we performed a coculture of B16-OVA tumor cells and OT-I T cells. We found VC treatment in the coculture system enhanced T cell activation, as determined by the increased expression of CD69, a marker for T cell activation downstream of TCR signaling, in the WT, but not TET2-KO, B16-OVA clones (Figure 5C). In addition to the in vitro coculture system, we also observed increased CD69 expression on T cells in the B16-OVA syngeneic tumor model following VC treatment (Figure 5, D and E). Moreover, VC treatment also increased OT-I T cell killing in the WT B16-OVA coculture system, but not the TET2-KO group, as quantified by live tumor cell counts (Figure 5, F and G). These data demonstrate that VC increases tumor antigen presentation, which is associated with T cell activation and tumor killing ability largely dependent on TET2 expression in tumor cells.

### TET2 expression in the tumor cells is essential for activation of tumor-infiltrated T cells and establishing IFN-γ–regulated tumor communication.

**Figure 6:**
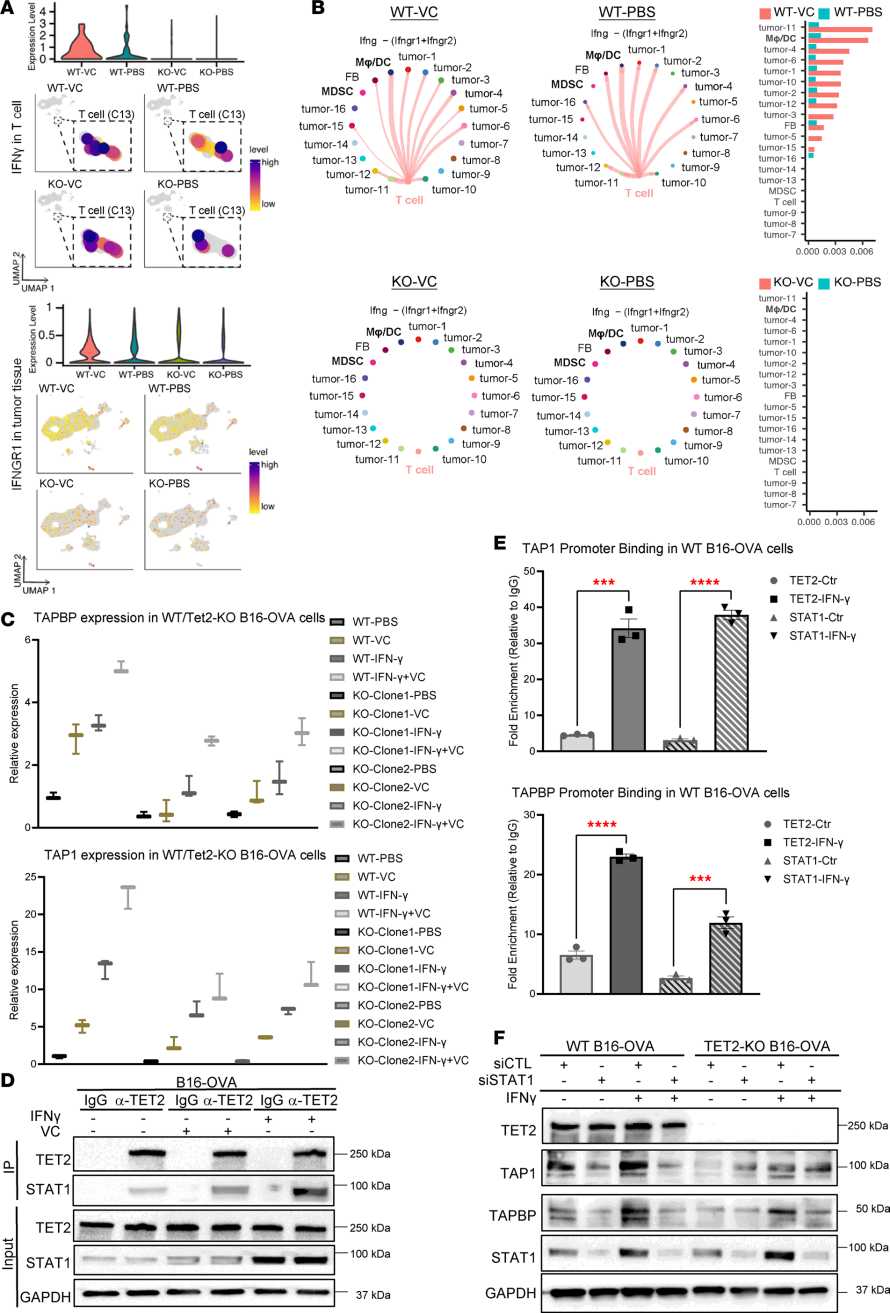
TET2 coordinates IFN-γ expression and its intercellular signaling, which is augmented by VC treatment. (**A**) Single-cell expression of IFN-γ and its receptor IFNGR1 was summarized in the UMAP plot and quantified in the violin plot above. (**B**) Intercellular communication of the IFN-γ signal between the T cell population and tumor clusters in the WT or TET2-KO tumor microenvironment treated with PBS control or VC are presented in the network plot; each line indicates an IFN-γ signal communication between 2 populations and the strength of communication was quantified in the bar plot. (**C**) The expression of key MHC I antigen-presenting genes TAP1, TAPBP, and B2M in the WT B16-OVA cells treated with IFN-γ and VC was determined by qPCR. Data represented as mean ± SD, with 3 replicates. (**D**) Coimmunoprecipitation of TET2 with STAT1 following treatment of B16-OVA cells with IFN-γ and/or VC. (**E**) The promoter region binding of TET2 and STAT1 on the TAP1 and TAPBP genes was analyzed through a ChIP assay. (**F**) Effects on TAP1 and TAPBP expression in B16-Ova (WT and KO) cells following STAT1 knockdown with siRNA, or with control (CTRL) siRNA. Data represented as mean ± SD, with 3 replicates. *P* values were calculated by unpaired, 2-tailed Student’s *t* test, with multiple comparisons corrected using Bonferroni’s method. ***P < 0.001, ****P < 0.0001.

The expression of MHC class I antigen presentation genes in tumor cells has been shown to be activated by IFN-γ mostly produced from the tumor-infiltrated T cells (50, 51). To address how antigen presentation genes are activated in the B16-OVA melanoma model, we performed an “intercellular communication” analysis (52) by matching the expression of ligand-receptor pairs in the scRNA-seq dataset. This approach analyzes intercellular communication networks derived from scRNA-seq data, classifying signal pathways and patterns. Importantly, we found the T cell cluster (cluster 13) was the major source of IFN-γ within the tumor microenvironment, while the IFN-γ receptor (IFNGR1) was expressed in each of the clusters (Figure 6A). VC treatment enhanced IFN-γ expression in T cells and IFNGR1 in tumor cells, which was dependent on the expression of TET2 in tumors (Figure 6A). Consistent with this, we observed IFN-γ signal communication between the T cell cluster and various tumor clusters, including clusters 0–5 in the WT groups. Additionally, other cell types, including the macrophage/dendritic cell cluster (cluster 9) and the fibroblast cluster (cluster 15), were also found to be responsive to IFN-γ produced by the tumor-infiltrated T cells. VC treatment was shown to increase the IFN-γ–regulated intercellular communication between the infiltrated T cells and its target clusters (Figure 6B). Notably, the intercellular communication of the IFN-γ response pathway was reduced in the TET2-KO groups, and slightly enhanced in TET2 WT cells without VC treatment (Figure 6B), paralleling expression of IFN-γ in the infiltrated T cells (Figure 6A). These results indicate that TET2 expression in tumor cells is important for the activation of tumor-infiltrated T cells to produce IFN-γ, leading to responses in tumor cells and other cells of the tumor microenvironment that are robustly induced with VC treatment.

### TET2 directly regulates the expression of certain antigen presentation genes.

We next addressed whether TET2 directly regulates the expression of antigen presentation genes. As shown in Figure 6C, VC enhanced the expression of MHC I antigen-presenting genes TAP1 and TAPBP in WT B16-OVA tumor cells. The effect of VC was reduced in the TET2-KO tumor cells, indicating that the increased expression of antigen presentation genes is partly dependent on TET2 (and see below). IFN-γ has been reported to induce expression of some antigen presentation genes, including TAP1 and TAPBP (49, 50, 53). We treated WT or TET2-KO B16-OVA cells with IFN-γ and observed increased expression of these antigen presentation genes, while VC further enhanced their expression when combined with IFN-γ (Figure 6C). IFN-γ has been shown to activate the JAK/STAT pathway to promote target gene transcription through activated STAT transcription factors (54). Our previous study showed that IFN-γ stimulated the binding between TET2 and STAT1 to activate target gene transcription, including those encoding chemokines (38), and we show here that in B16 melanoma cells INF-γ stimulates the interaction between TET2 and STAT1 (Figure 6D). Additionally, using ChIP assays, we found that STAT1 and TET2 bind to the promoter regions of the TAP1 and TAPBP genes following IFN-γ treatment of the B16 cells (Figure 6E). Immunoblotting shows that TAP1 and TAPBP expression is basally reduced with TET2 KO and that induction by IFN-γ is impaired, but not eliminated, with TET2 KO (Figure 6F). Therefore, we analyzed expression of TET1, -2, and -3 in B16 melanoma and CT-26 tumors and found that TET2 was expressed at the highest level, with TET3 at intermediate levels and TET1 at the lowest level (Supplemental Figure 5A). Interestingly, TET2 KO as well as TET3 KO reduced basal and IFN-γ–induced expression of TAPBP (Supplemental Figure 5B), indicating critical roles for multiple TET proteins in control of certain antigen expression genes. However, TET2 KO only partly suppressed IFN-γ–induced B2M expression, while TET3 had no effect on B2M expression (Supplemental Figure 5B), suggesting that other factors are involved in B2M expression. Nevertheless, ChIP analysis revealed that TET2 interacts with the B2M promoter following IFN-γ treatment (Supplemental Figure 5C), potentially explaining the induction of B2M by VC treatment. Consistent with the relative levels of expression of TET2 and TET3, TET2 KO led to a larger reduction in 5hmC in B16 cells than that observed with TET3 KO (Supplemental Figure 5D).

### The expression of antigen-presenting genes correlates with cancer patient outcomes and TET activity.

**Figure 7:**
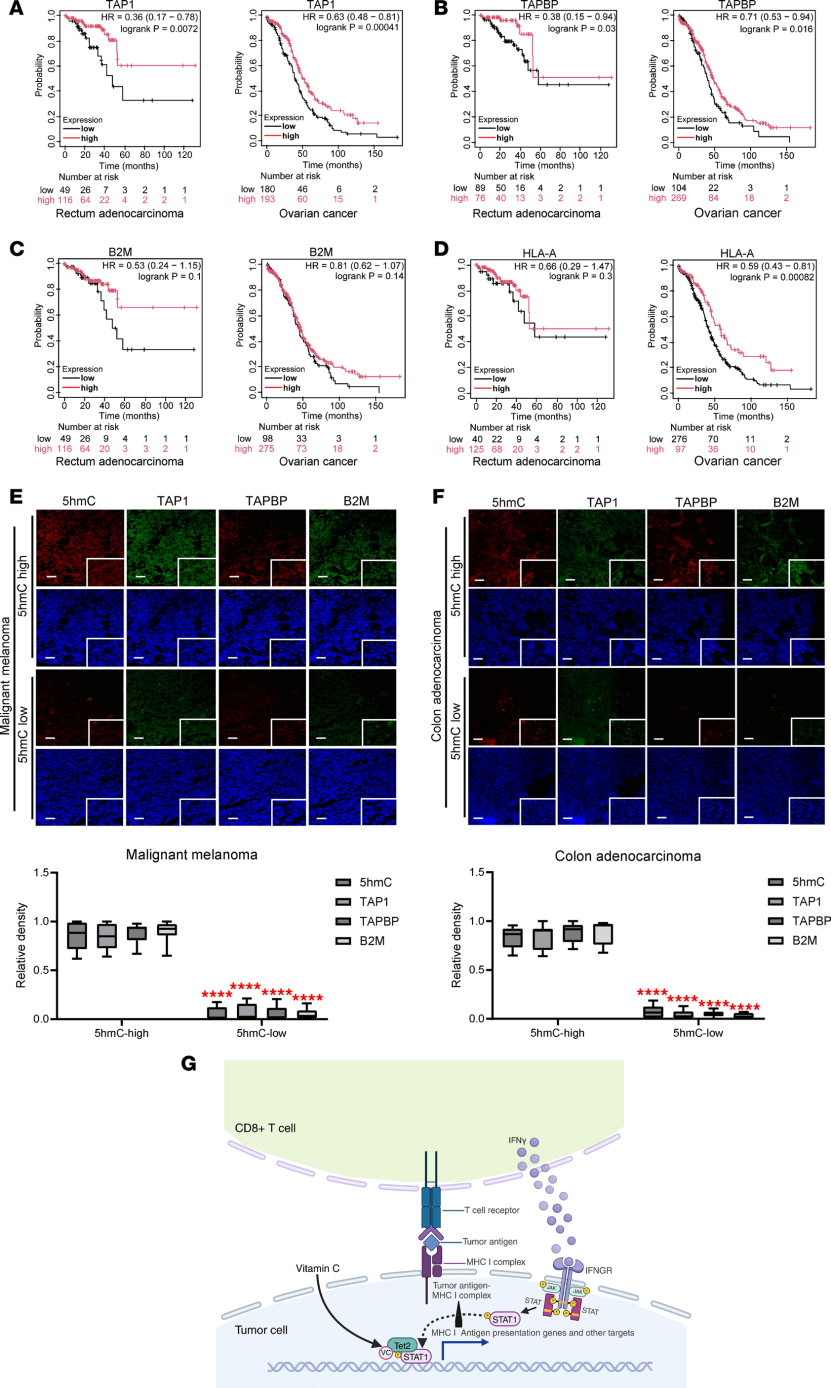
The expression of antigen presentation genes correlates with better prognosis and higher TET activity in human patients. The expression level of key MHC I antigen presentation genes TAP1 (**A**), TAPBP (**B**), B2M (**C**), and HLA-A (**D**) and their correlation with patient survival predictions were summarized in different human cancer types. Data collected from the Kaplan-Meier Plotter database, with HR indicating hazard ratio and logrank P indicating *P* value. Human cancer tissue was purchased from Biomax (Methods) and stained for 5hmC, TAP1, TAPBP, B2M, and with DAPI for immunofluorescence imaging in malignant melanoma (**E**) and colon adenocarcinoma (**F**). Scale bars: 100 μm. White boxes show ×20 magnification. The relative density of 5hmC, TAP1, TAPBP, and B2M relative to DAPI was calculated from at least 6 replicates under enlarged fields. Unpaired, 2-tailed Student’s *t* test with multiple comparisons corrected using Bonferroni’s method was used to determine the *P* value of positive cells between 5hmC-high samples and 5hmC-low samples. ****P < 0.0001. (**G**) Schematic model of the role of TET2 downstream of IFN-γ signaling and VC in regulating tumor antigen presentation and T cell activity directed to the tumor cell.

To examine whether expression of antigen presentation genes correlates with patient survival, we collected survival data for human cancer patients from the Kaplan-Meier Plotter database. The levels of expression of antigen expression genes TAP1, TAPBP, B2M, and HLA-A (the human ortholog of the mouse H2-D1 gene) were shown to predict longer patient survival in colon and ovarian cancers (Figure 7, A–D). This is consistent with other studies showing that expression of antigen presentation genes correlates directly with patient survival (11–13). Next, we obtained multiple human malignant melanoma and colon adenocarcinoma tissue samples and stained for 5hmC as a marker for TET2 activity (55) and for the key MHC I antigen-presenting genes TAP1, TAPBP, and B2M. We then grouped these human tumor samples into a 5hmC-high class or a 5hmC-low class depending on the overall 5hmC level expressed in tumor tissue. As shown in Figure 7E, expression of TAP1, TAPBP, and B2M was higher in 5hmC-high melanoma samples than 5hmC-low samples, indicating a positive correlation between TET2 activity and MHC I antigen-presenting genes. Similarly, there was a positive correlation between TET2 activity and these 3 MHC I antigen-presenting genes in colon adenocarcinoma patients (Figure 7F). Together, these results indicate a direct correlation between TET activity and antigen presentation machinery gene expression and a better overall prognosis.

## Discussion

MHC class I–controlled antigen presentation is a critical biological process found in all nucleated cells in vertebrates, including tumor cells. Increased antigen presentation by tumor cells promotes T cell–directed tumor cell killing and, consistently, reduced tumor antigen presentation is one mechanism for escape from immune surveillance (4, 5). Loss of expression of certain antigen presentation genes occurs in many cancer types, with a range from around 30% in renal cancer to over 85% in thyroid cancer, which is explained through gene mutations and through epigenetic silencing (15). Enhancing the MHC class I expression response promotes antitumor immunity directed by T cells (10–13). These findings place reduced antigen gene expression among other key mechanisms such as poor T recruitment, T cell exhaustion, loss of critical IFN-γ signaling, and an immunosuppressive tumor microenvironment that are associated with reduced tumor immunity and immunotherapy responses. Mechanisms to enhance tumor immunity, based on stimulating T cell activity, include immune adjuvants, tumor-directed vaccines, and oncolytic viruses (4).

TET proteins (TET1, -2, -3) are critical regulators of gene expression, functioning to demethylate DNA regulatory regions through interactions with certain transcription factors (19–22). TET proteins utilize α-KG as a required cofactor and are known to be activated by VC (22, 23). Recently, our group and others showed direct interaction between VC and TET2, which promotes catalytic activity (36, 37). TET2 is frequently mutated in lymphoid and myeloid malignancies (26–28); however, in solid tumors loss-of-function mutations in TET proteins are not commonly observed, yet TET activity is frequently reduced (29–31). Using the B16-OVA melanoma model, we previously demonstrated that TET2 is important for tumor-associated IFN-γ–regulated expression of chemokines CXCL9, -10, and -11. Loss of TET2 expression in the B16 tumor model led to reduced T cell recruitment, while stimulation of tumors with VC promoted T cell recruitment and enhanced response to checkpoint inhibitor therapy (38). Consistently, it was shown in an IDH-mutant cholangiocarcinoma model that reduced TET2 activity, via loss of α-KG, leads to reduced T cell infiltration. IDH inhibitor treatment restored T cell recruitment, enhanced the IFN-γ response, and promoted the therapeutic response to checkpoint inhibitor therapy (41).

Here, we show that TET2 in B16-OVA melanoma tumor cells is important for expression of some MHC class I antigen presentation genes, including the key regulators TAP1 and TAPBP, which are regulated downstream of IFN-γ–induced signaling. scRNA-seq analysis of cells isolated from B16-OVA melanoma tissue indicated VC treatment upregulates the antigen presentation pathway and associated genes in tumor cells in a TET2-dependent manner (Figure 2, Figure 3, A and C, and Figure 4), which were broadly not upregulated in WT tumors versus TET2-KO tumors without VC (Supplemental Figure 3E, and see below), which is likely due to relatively low levels of IFN-γ production by T cells in the B16 model (Figure 6A). To address the mechanism whereby TET2 regulates expression of some of these genes, we first showed that IFN-γ induces an interaction between TET2 and STAT1 and that TET2 and STAT1 subsequently associate with the TAP1 and TAPBP promoters (Figure 6, D and E). While TET2 ChIPs at the B2M promoter (Supplemental Figure 5C), its loss only weakly reduces B2M expression in B16 cells and does not reduce B2M tumor expression as measured through flow cytometry (Figure 5B), indicating a role for additional actors in promoting B2M gene expression. Nevertheless, VC treatment promotes B2M levels as measured through flow cytometry in a T cell–dependent manner (Figure 5B). We noticed that TET2 KO did not fully inhibit baseline or VC-induced expression of some antigen presentation genes (see Figure 6C). Thus, we explored the potential involvement of TET3, which is expressed in the tumor models that we studied (Supplemental Figure 5A). Interestingly, we found that TET3 KO in the B16 cells led to reduced basal and VC-induced expression of TAPBP, but had no effect on B2M expression (Supplemental Figure 5B). Thus, both TET2 and TET3 appear to contribute to the regulation of certain antigen presentation genes and to their enhancement of expression by VC. Consistent with data presented here, it was found that TET2 mutation in murine B cells leads to hypermethylation at the antigen presentation gene TAPBP and some H2 genes (56).

The results described above place TET2 and TET3 as critical transcriptional regulators associated with the IFN-γ–stimulated response, particularly focused on the regulation of certain antigen presentation pathway genes, but also contributing to control of other genes and processes. Using intercellular communication analysis from the scRNA-seq data, we show that loss of TET2 expression in the B16 tumors leads to loss of IFN-γ expression in T cells, which then reduces IFN-γ–induced responses in the tumor and tumor microenvironment (Figure 6, A and B). Tumor-associated roles for TET2 in enhancing T cell responses presumably include antigen presentation as well as other mechanisms such as production of key chemokines (38). Thus, TET2 (and potentially TET3) initiates a feed-forward regulatory loop leading to T cell–derived IFN-γ production, and corresponding downstream TET2 activity that is strongly promoted by VC (see model, Figure 7G).

Higher expression levels of MHC class I antigen presentation gene expression correlates with better patient outcomes (Figure 7, A–D) presumably based on enhanced antitumor immunity. Others have found expression of antigen presentation genes correlates directly with patient outcomes (11–13). To correlate TET activity with antigen presentation gene expression, we quantified 5hmC in tumor tissue samples as a marker of TET activity (55) and found that low 5hmC tumor levels correlated with lower expression of some antigen presentation genes (Figure 7, E and F). These results support the hypothesis that poor antigen presentation in tumors is based partly on loss of tumor-associated TET activity. Mechanisms to explain loss of TET2 activity in cancer are not well established, but include reduction of α-KG levels in some cancers (30) as well as loss of TET2 expression (31). Since TET activity is stimulated by T cell–derived IFN-γ, loss of T cell expression of IFN-γ would lead to reduced tumor-associated TET activity (see model, Figure 7G).

Some studies have linked VC therapy with better cancer outcomes, and a variety of mechanisms have been proposed to explain the results, including VC-induced oxidative stress and inhibition of HIF transcription factor activity, along with direct cytotoxicity to tumor cells (33, 42, 57, 58). The results presented here and previously by our group (36, 38, 56) indicate that TET2 is directly activated by VC to stimulate expression of genes involved in immune signaling and in T cell recruitment. Additionally, it was reported that VC activates a TET2/NF-κB axis in dendritic cells to promote immunogenic properties of these cells (59). Thus, the effects of VC on tumor immunity as related to antigen presentation may occur in professional antigen-presenting cells. In this regard, we hypothesize that some in vivo anticancer effects of VC treatment are related to promotion of tumor immunity, which is TET dependent (see model, Figure 7G). Supported by the animal tumor studies (see Figure 1), we hypothesize that the antitumor effects of VC will be most effective in combination with immune checkpoint inhibitor therapy.

## Methods

### Sex as a biological variable.

Our study examined male and female animals, and similar findings are reported for both sexes.

### Reagents.

VC (L-ascorbic acid) was purchased from Sigma-Aldrich and diluted in PBS to a 1 M stock followed by 0.25-μm filtration for sterilization before use in cell culture (at a final concentration of 500 μM) or mice (1 g/kg for i.v. injection or other doses as indicated). Recombinant IFN-γ was purchased from R&D Systems, diluted in PBS, and used at 100 ng/mL in cell culture.

### Cell lines.

B16-OVA (B16F10 cells expressing ovalbumin) cells were purchased from ATCC and cultured at 37°C and 5% CO_2_ in DMEM (Corning) containing 10% FBS (VWR Life Sciences, Seradigm) and penicillin (5 U/mL, Sigma-Aldrich). CT-26 cells were purchased from ATCC and cultured at 37°C and 5% CO_2_ in RPMI (Corning) containing 10% FBS and penicillin.

### Human tissue microarrays.

Human malignant melanoma, colon adenocarcinoma and their corresponding healthy tissue sections were purchased from Biomax (ME241b, T382c, CO243b).

### Antibodies.

The following antibodies were used in this study: GAPDH (GeneTex, 110118), IgG (Cell Signaling Technology [CST], 3900S), TET1 (Abcam, ab191698), TET2 (CST, 18950S), TET3 (CST, 99980S), NF-κB (p65) (CST, 8242S), STAT1 (CST, 9172S), 5hmC (Active Motif, 39769), 5mC (Active Motif, 61479), 5hmC (CST, 51660S), B2M (CoraLite 488, Proteintech, CL488-13511), TAPBP (LS Bio, LS-B9746-GOS10-100), TAP1 (Proteintech, 11114-1-AP), CD3 Alexa Fluor 700 (Invitrogen, MHCD0324), CD4 PE-Cyanine7 (Invitrogen, MHCD0412), CD8 PE-Alexa Fluor 610 (Invitrogen, MHCD0822), CD69 Super Bright 645 (eBioscience, 64-0691-82), CD25 Bright 436 (eBioscience, 62-0251-82), CD11c FITC (eBioscience, 11-0114-82), and PE-anti–mouse H-2Kb bound to SIINFEKL (Invitrogen, 12-5743-82).

### Gene KO by CRISPR/Cas9 system.

TET2-KO and TET3-KO B16-OVA and TET2-KO CT-26 cells were generated through the CRISPR/Cas9 system by the transient CRISPR strategy (44). Cells were transiently transfected with a Cas9 and single-guide RNA (sgRNA) plasmid with EGFP expression (PX458; Addgene plasmid 48138). Following transfection for 2 days, single cells were sorted by FACS based on EGFP expression into 96-well plates. KO clones were validated by Western blotting with TET2 or TET3 antibody and DNA sequencing. PCR primers used for amplifying the sgRNA-targeted sequence from genomic DNA and the gRNA sequence used for targeting TET2/3 are summarized in Table 1.

### Western blot analysis.

Cultured cells were washed with PBS twice and then lysed in a 1× Laemmli sample buffer (Bio-Rad) directly. Lysates were heated at 100°C for 10 minutes and centrifuged at 18,000*g* for 10 minutes before loading in 4%–15% precast gels (BIio-Rad). Samples were electrophoresed at 80 V for 15 minutes, then switched to 150 V for another 1 hour, and then transferred to PVDF membranes at 100 V for 90 minutes. Membranes were then blocked in a 5% milk/TBST for 1 hour at room temperature, followed by primary antibody incubation overnight at 4°C. HRP-conjugated secondary antibody was then applied and incubated for 2 hours at room temperature before washing with PBST 5 times. Membranes were imaged by a ChemiDoc MP Imaging system (Bio-Rad) and organized with Image Lab software before washing with PBST 5 times.

### In vivo tumor progression and immunotherapy models.

B16-OVA cells or CT-26 cells (2 × 10^5^) were transplanted s.c. into the right back flanks of 6-week-old male and female C57BL/6J or BALB/c mice, respectively (The Jackson Laboratory) in a cold PBS suspension to form tumors in relevant syngeneic models. Tumor size was measured with a caliper every 2 days, and tumor volume was calculated by width^2^ × length/2. Mice were sacrificed when tumors reached the maximum allowed size (20 mm in diameter). Tumor weight was also recorded every 2 days to monitor mouse health; mice were sacrificed when their body weight loss was greater than 20%.

For the anti–PD-L1 immunotherapy studies, mice were injected i.p. with 200 μg anti–PD-L1 (clone 10F.9G2, BP0101, BioXCell) 3 times per week for 2 weeks, starting 1 week after tumor implantation to ensure successful tumor growth. For VC treatment, mice were injected i.v. with sodium ascorbate (1g/kg) or PBS every day at indicated periods or otherwise indicated in the figures. Mice were monitored for tumor growth every 2 days and sacrificed when tumors reached 20 mm in diameter. Statistical analysis was conducted using GraphPad Prism 9 software. Kaplan-Meier curves and corresponding log-rank (Mantel-Cox) test were used to evaluate the statistical differences between groups in survival studies.

### RNA purification, quantitative PCR.

For quantitative PCR (qPCR), total RNA was purified from cells or mouse tumor samples using RNeasy Plus Mini Kit (QIAGEN) according to the manufacturer’s instructions. cDNA was synthesized with 1 μg of RNA using SuperScript III First-Strand Synthesis System (Invitrogen). qPCR was performed in triplicate using SYBR Green PCR Master Mix (Applied Biosystems) in a QuantStudio 6 Flex Real-Time PCR System (Applied Biosystems). All primers for qPCR are listed in Table 2.

### scRNA-seq.

Tumor tissues from WT or TET2-KO B16-OVA syngeneic tumor model treated with VC or PBS were harvested and dissected. One gram of tumor tissue for each sample was used and cut into approximately 1 mm^3^ pieces with scissors and tweezers on ice, followed by 30-minute incubation of 20 mL digestion buffer composed of DMEM media supplemented with 1 mg (100 U)/mL DNase I (Sigma-Aldrich) and 0.2 mg/mL Collagenase/Dispase (Roche) at 37°C. Digested cell suspension was then filtered through sterile 40-μm nylon mesh to make single-cell suspensions. Then, single-cell suspensions were centrifuged at 200*g* for 5 minutes at 4°C and rinsed in 10 mL FACS buffer (sterile PBS, 2% FBS, 0.05% sodium azide, and 2 mM EDTA). After centrifuged again at 1200 rpm for 5 minutes at 4°C, cell pellets were rinsed in 5 mL 1× RBC lysis buffer (Invitrogen) and incubated on ice for 3 minutes to remove red blood cells. Another 20 mL FACS buffer was used to top up the cell suspension before centrifugation at 1200 rpm for 5 minutes at 4°C. Cell pellets were then washed twice before resuspending in 10 mL FACS buffer and then counted using trypan blue (Sigma-Aldrich) staining and a TC20 automated cell counter (Bio-Rad). Dead cells from single-cell suspensions were then removed by Dead Cell Removal Kit (Miltenyi Biotec) via positive labeling and selection of dead cells using magnetic separation. Additional rounds of dead cell removal were performed to achieve the goal of more than 90% live cell counts in our single-cell suspensions. Subsequently, 10,000 single cells from each sample were then collected and used for scRNA-seq at 10× Genomics.

Each condition for scRNA-seq was conducted in triplicate. Chromium Next GEM Single Cell 3′ GEM, Library & Gel Bead Kit v3.1 (10× Genomics) was used for library preparation and the Illumina NextSeq 2000 system was used for sequencing at the 10× Genomics Sequencing Facility at the University of North Carolina - Chapel Hill (UNC-CH). Paired-end FASTQ sequences were aligned to the mouse genome (GRCm38/mm10). We carried out quality control using Seurat 4.1.1 (60). We utilized LIGER (61) (version 1.0.0) for sample integration, normalization, clustering, and visualization as dedicated by the package. Gene expression count data were normalized by dividing gene counts of each cell by the total count of that cell, multiplied by 10,000 and log_2_ transformed. We performed integrative non-negative matrix factorization and quantile alignment for each dataset and cluster. Two-dimensional visualization and clustering were carried out with resolution set as 0.30 and distance as cosine, nearest neighbors set to 30, and minimum distance to 0.30. To identify the DEGs among clusters, we performed 2-sided nonparametric Wilcoxon’s rank-sum test with Benjamini-Hochberg correction. The top 100 DEGs were selected for comparison and were used for cluster annotation. We assigned cell subtypes based on previously characterized molecular markers (62) and tumor cells were defined by their expression of melanocyte-specific genes, including *Mlana*, *Tyrp1*, *Mc1r*, *Dct*, *Pmel*, and *Gpnmb*. Characterization of the groupings from cluster 9 was derived from dendritic cell and macrophage markers, as described previously (63).

### GO enrichment analysis.

ClusterProfiler 4.0 (64) was used to conduct gene ontology enrichment analysis, elucidating underlying biological phenomena in the 4 groups: WT-VC, WT-PBS, KO-VC, and KO-PBS. The approach was made at a cell population level and at an individual cluster level. With an FDR-adjusted *P*-value significance threshold of 0.05, enriched GO terms associated with biological process were based on the top 100 DEG list.

### Single-cell intercellular communication inference.

We employed CellChat (52) to study intercellular signaling networks among various cell types for analysis of the immune response in the tumor microenvironment. CellChatDB for mouse, a database of 2,021 validated molecular interactions containing the ligands, receptors, and their cofactors, was used as the basis for the ligand-receptor analysis (intercellular communication) under the standard condition.

### Flow cytometry.

Tumor tissue was harvested and dissected to generate single-cell suspensions as described in *scRNA-seq* above without the dead cell removal steps. Then, cells from tumor tissue were resuspended in FACS buffer and incubated with Fc blocker for 10 minutes on ice. After washing with FACS buffer, the cells were incubated with fluorescent antibodies for 20–30 minutes and washed 3 times with staining buffer before measurement. An Attune NxT acoustic focusing cytometer (AFC2, Invitrogen) was used to collect data, which were analyzed using FlowJo (10.6.2) software (BD Biosciences). The following antibodies and reagent were used: CD3 Alexa Fluor 700, CD4 PE-Cyanine7, CD8 PE-Alexa Fluor 610, CD69 Super Bright 645 (SB645), CD45 eFluor 660, cell viability solution (7-aminoactinomycin D, 7-AAD, Thermo Fisher Scientific), CD25 Bright 436, CD11c FITC, PE-anti-mouse H-2Kb bound to SIINFEKL, and B2M.

### Immunofluorescent staining.

For multiple-fluorescence staining, the paraffin-embedded slides were initially dewaxed with xylene, followed by antigen retrieval achieved through boiling for 30 minutes in 10 mM citrate buffer (pH 6.0). Subsequently, the sections were permeabilized with 0.25% (v/v) Triton X-100 for 20 minutes and then blocked with 5% goat serum in PBS at room temperature for 60 minutes. For 5hmC staining, an additional treatment with 2N HCl for 15 minutes at room temperature was performed. Primary antibodies were applied onto the slides and left to incubate overnight at 4°C, followed by incubation with fluorophore-conjugated mouse or rabbit secondary antibodies for 60 minutes at room temperature. Finally, the slides were stained with DAPI for 5 minutes at room temperature and mounted in ProLong Gold Antifade Mountant (Invitrogen). Images were captured using an FV1000 confocal microscope (Olympus).

### B16-OVA and OT-I coculture.

WT or TET2-KO B16-OVA cells were cultured in 6-well plates and grown to approximately 50% confluence followed by EGFP plasmid (pcDNA3-EGFP, Addgene) transfection with Lipofectamine 3000 reagent (Invitrogen) before OT-I T cell coculture. OT-I CD8^+^ T cells and dendritic cells were isolated from OT-I mice obtained from The Jackson Laboratory [C57BL/6-Tg (Tcra, Tcrb)1100Mjb/J] using the CD8a^+^ T cell Isolation Kit, mouse (Miltenyi Biotec) and Pan Dendritic Cell Isolation Kit, mouse (Miltenyi Biotec). Isolated OT-I T cells (1 × 10^6^/sample) and dendritic cells (1 × 10^5^/sample) were then added to B16-OVA tumor cells with or without 500 μM VC treatment for 24 hours (65). Then, tumor cells were imaged and counted under the microscope.

### ChIP-qPCR.

ChIP assay was performed using the ChIP-IT Express Chromatin Immunoprecipitation Kits (Active Motif), following the manufacturer’s instructions. B16-OVA tumor cells were treated with PBS, VC, IFN-γ, and VC plus IFN-γ for 24 hours; then, cells were harvested and fixed with fixation buffer (containing 1% formaldehyde). Cross-linked cells were washed with cold PBS and then resuspended in ChIP buffer supplemented with protease inhibitor and PMSF. Next, the cells were homogenized, and the chromatin sheared to approximately 200- to 600-bp fragments by using a Covaris Sonicator for 15 minutes (25% power) at 4°C. A 500-μL volume (100 μg chromatin mixture) was used per immunoprecipitation, and 5 mL (1% of total) was kept as the input DNA. As a negative control, rabbit or mouse nonimmune IgG was used (Thermo Fisher Scientific). To detect TET2, RELA, or STAT1 bound to the promoters, rabbit anti-TET2 (CST) and rabbit anti-STAT1 (CST) were used. For the detection of ChIP-generated DNA, real-time PCR was performed by the SYBR GREEN PCR Master Mix (Applied Biosystems), and the primers are listed in Table 3.

### Statistics.

Data analysis was performed using GraphPad Prism software. Normally distributed data were analyzed using an unpaired, 2-tailed Student’s *t* test, and multiple comparisons were corrected using Bonferroni’s method; a log-rank (Mantel-Cox) test was used for the mouse survival assays. Two-sided Wilcoxon’s rank-sum tests were used to identify DEGs. Statistical significance was defined as an adjusted *P* value (Bonferroni’s correction) of less than 0.05.

### Study approval.

All animal experiments were approved by the IACUC at the UNC-CH. For human samples, all tumor microarrays were purchased from Biomax (see above).

### Data availability.

The scRNA-seq datasets generated in this study are available in the NCBI Gene Expression Omnibus (GEO GSE227998). All raw data are available as a Supporting Data Values Excel file, with tabs for each applicable figure panel.

## Author contributions

MC, AC, JDW, QZ, NB, WFM, JM, NB, YX, and AB conceptualized the study. MC, AC, ZJL, SY, and MS developed the methodology. MC, AC, and ZJL carried out the investigation. YX and AB acquired funding. MS and AB provided project administration. JDW and AB supervised the study. MC, AC, JM, YX, and AB wrote the manuscript.

## Supplementary Material

Supplemental data

Unedited blot and gel images

Supporting data values

## Figures and Tables

**Table 3:**
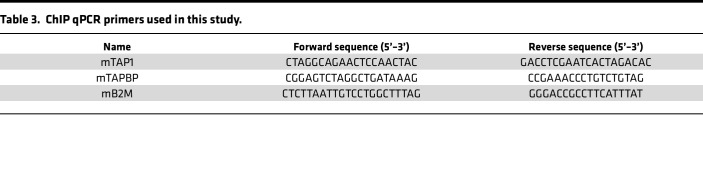
ChIP qPCR primers used in this study.

**Table 2:**
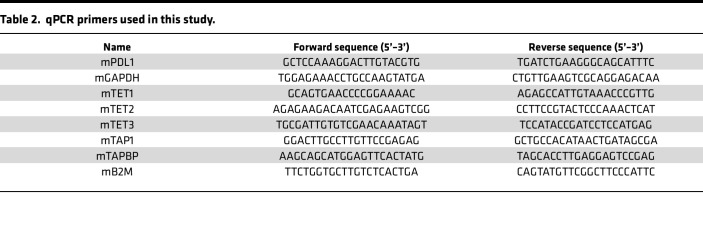
qPCR primers used in this study.

**Table 1:**
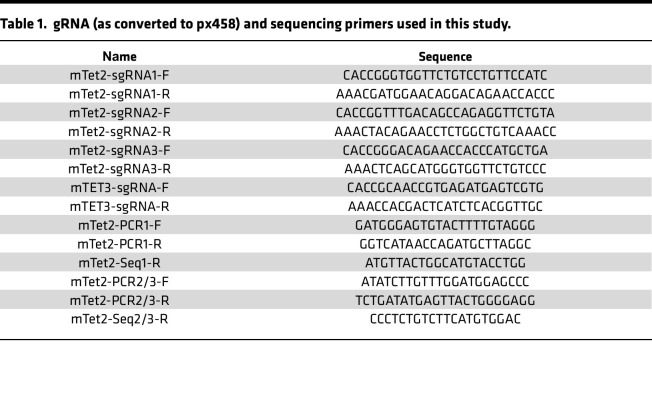
gRNA (as converted to px458) and sequencing primers used in this study.

## Version 1 10/10/2024

In-Press Preview

## Version 2 11/22/2024

Electronic publication
