# Supplementary material for: TET2 promotes tumor antigen presentation and T cell IFN-γ, which is enhanced by vitamin C
Source: JCI Insight. 2024 Nov 22;9(22):e175098. doi: 10.1172/jci.insight.175098 (PMC11601905; doi:10.1172/jci.insight.175098)
Supplement: Supplemental data [file jciinsight-9-175098-s078.pdf]

Figure S1

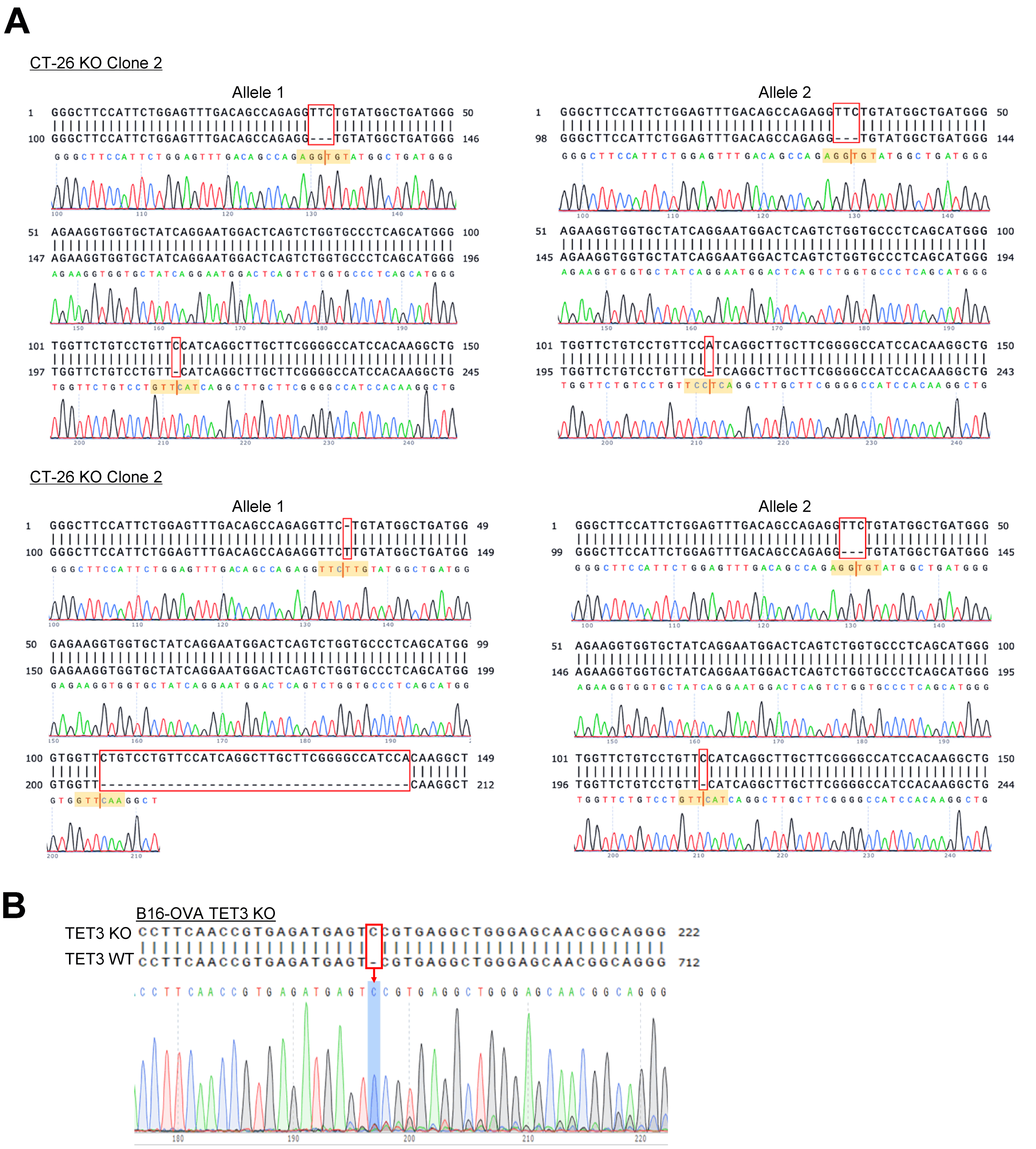
A
CT-26 KO Clone 2
Allele 1
Allele 2
CT-26 KO Clone 2
Allele 1
Allele 2
B
B16-OVA TET3 KO
TET3 KO
TET3 WT

Figure S2

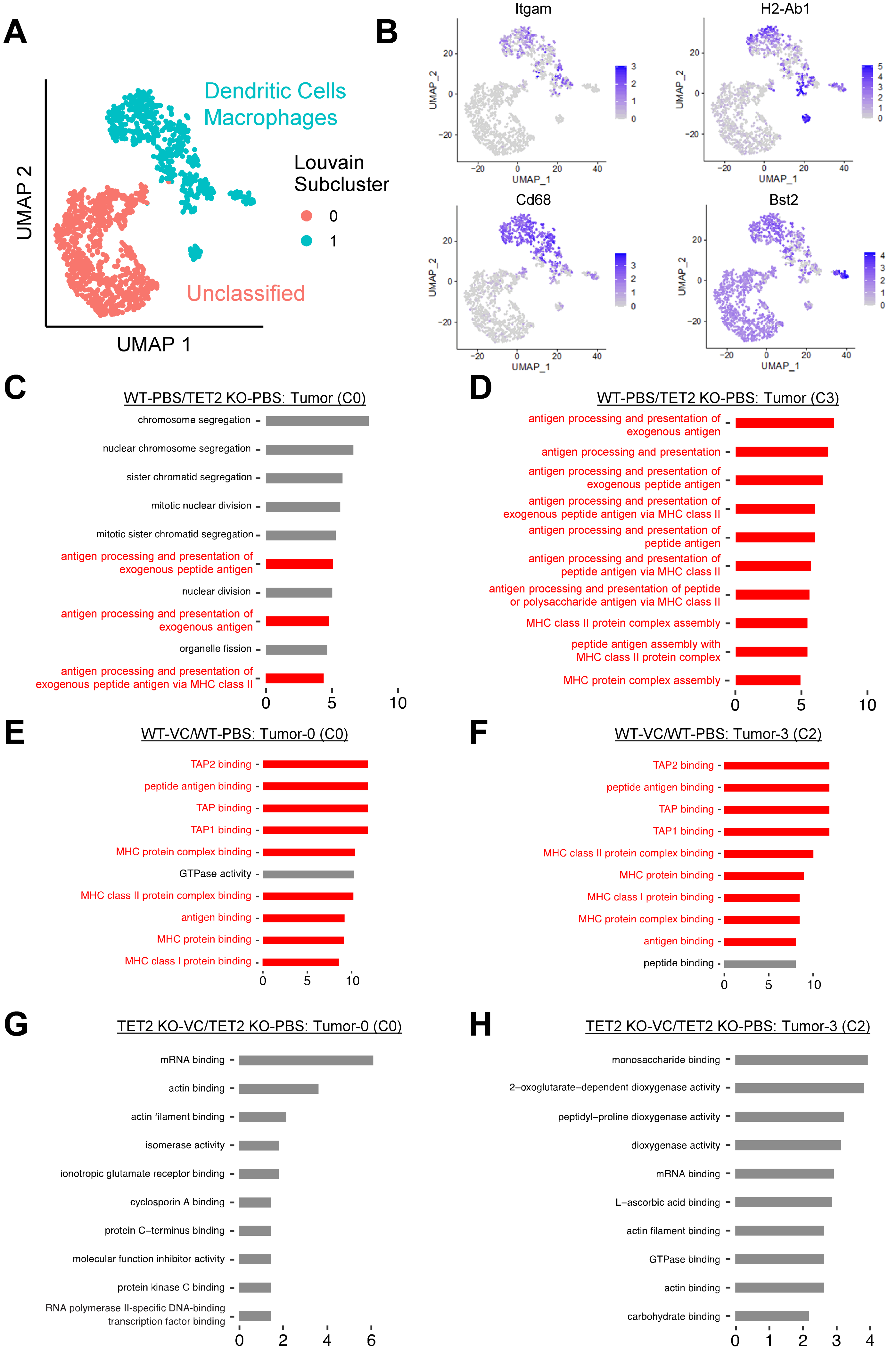

A
Dendritic Cells
Macrophages
Louvain Subcluster
0
1
Unclassified
UMAP 2
UMAP 1
B
Itgam
H2-Ab1
Cd68
Bst2
UMAP_1
UMAP_2
C
WT-PBS/TET2 KO-PBS: Tumor (C0)
chromosome segregation
nuclear chromosome segregation
sister chromatid segregation
mitotic nuclear division
mitotic sister chromatid segregation
antigen processing and presentation of exogenous peptide antigen
nuclear division
antigen processing and presentation of exogenous antigen
organelle fission
antigen processing and presentation of exogenous peptide antigen via MHC class II
D
WT-PBS/TET2 KO-PBS: Tumor (C3)
antigen processing and presentation of exogenous antigen
antigen processing and presentation
antigen processing and presentation of exogenous peptide antigen
antigen processing and presentation of exogenous peptide antigen via MHC class II
antigen processing and presentation of peptide antigen
antigen processing and presentation of peptide antigen via MHC class II
antigen processing and presentation of peptide or polysaccharide antigen via MHC class II
MHC class II protein complex assembly
peptide antigen assembly with MHC class II protein complex
MHC protein complex assembly
E
WT-VC/WT-PBS: Tumor-0 (C0)
TAP2 binding
peptide antigen binding
TAP binding
TAP1 binding
MHC protein complex binding
GTPase activity
MHC class II protein complex binding
antigen binding
MHC protein binding
MHC class I protein binding
F
WT-VC/WT-PBS: Tumor-3 (C2)
TAP2 binding
peptide antigen binding
TAP binding
TAP1 binding
MHC class II protein complex binding
MHC protein binding
MHC class I protein binding
MHC protein complex binding
antigen binding
peptide binding
G
TET2 KO-VC/TET2 KO-PBS: Tumor-0 (C0)
mRNA binding
actin binding
actin filament binding
isomerase activity
ionotropic glutamate receptor binding
cyclosporin A binding
protein C−terminus binding
molecular function inhibitor activity
protein kinase C binding
RNA polymerase II-specific DNA-binding transcription factor binding
H
TET2 KO-VC/TET2 KO-PBS: Tumor-3 (C2)
monosaccharide binding
2−oxoglutarate−dependent dioxygenase activity
peptidyl−proline dioxygenase activity
dioxygenase activity
mRNA binding
L−ascorbic acid binding
actin filament binding
GTPase binding
actin binding
carbohydrate binding

Figure S3

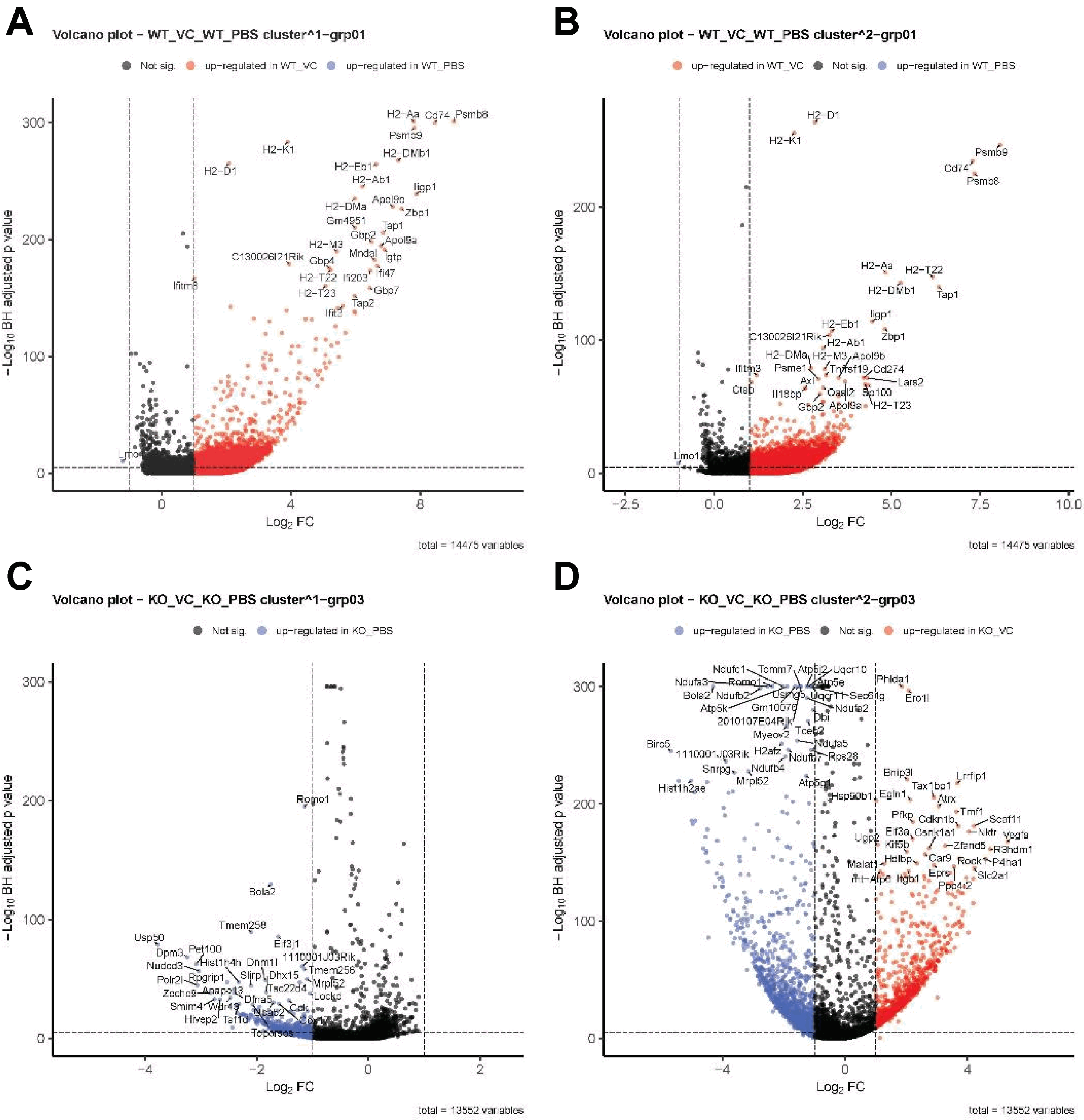
A
Volcano plot – WT_VC_WT_PBS cluster^1–grp01
Not sig.
up–regulated in WT_VC
up–regulated in WT_PBS
–Log10 BH adjusted p value
Log2 FC
total = 14475 variables
B
Volcano plot – WT_VC_WT_PBS cluster^2–grp01
up–regulated in WT_VC
Not sig.
up–regulated in WT_PBS
–Log10 BH adjusted p value
Log2 FC
total = 14475 variables
C
Volcano plot – KO_VC_KO_PBS cluster^1–grp03
Not sig.
up–regulated in KO_PBS
–Log10 BH adjusted p value
Log2 FC
total = 13552 variables
D
Volcano plot – KO_VC_KO_PBS cluster^2–grp03
up–regulated in KO_PBS
Not sig.
up–regulated in KO_VC
–Log10 BH adjusted p value
Log2 FC
total = 13552 variables

Figure S4

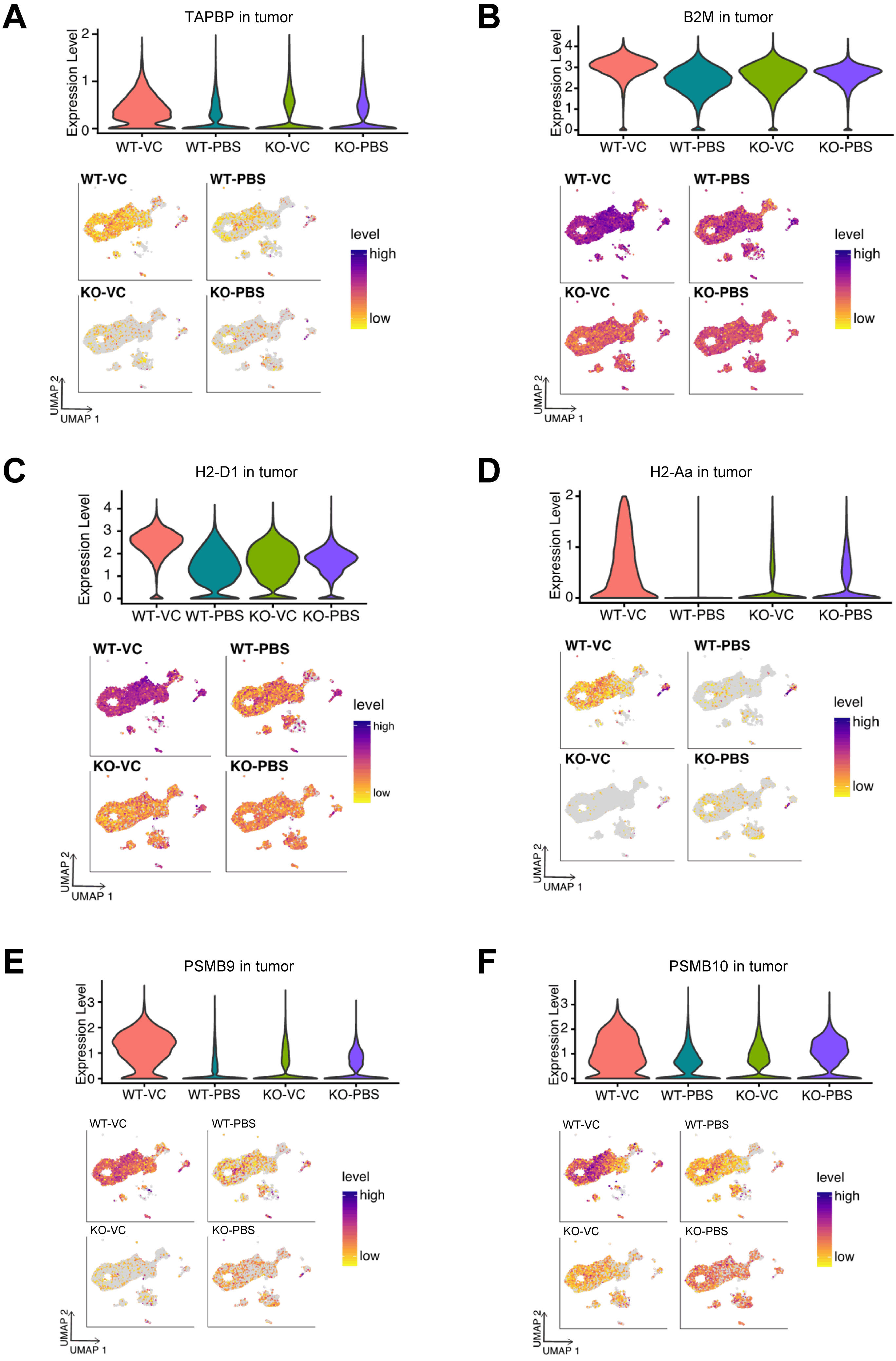
A
TAPBP in tumor
B
B2M in tumor
C
H2-D1 in tumor
D
H2-Aa in tumor
E
PSMB9 in tumor
F
PSMB10 in tumor
Expression Level
WT-VC
WT-PBS
KO-VC
KO-PBS
level
high
low
UMAP 1
UMAP 2

Figure S5

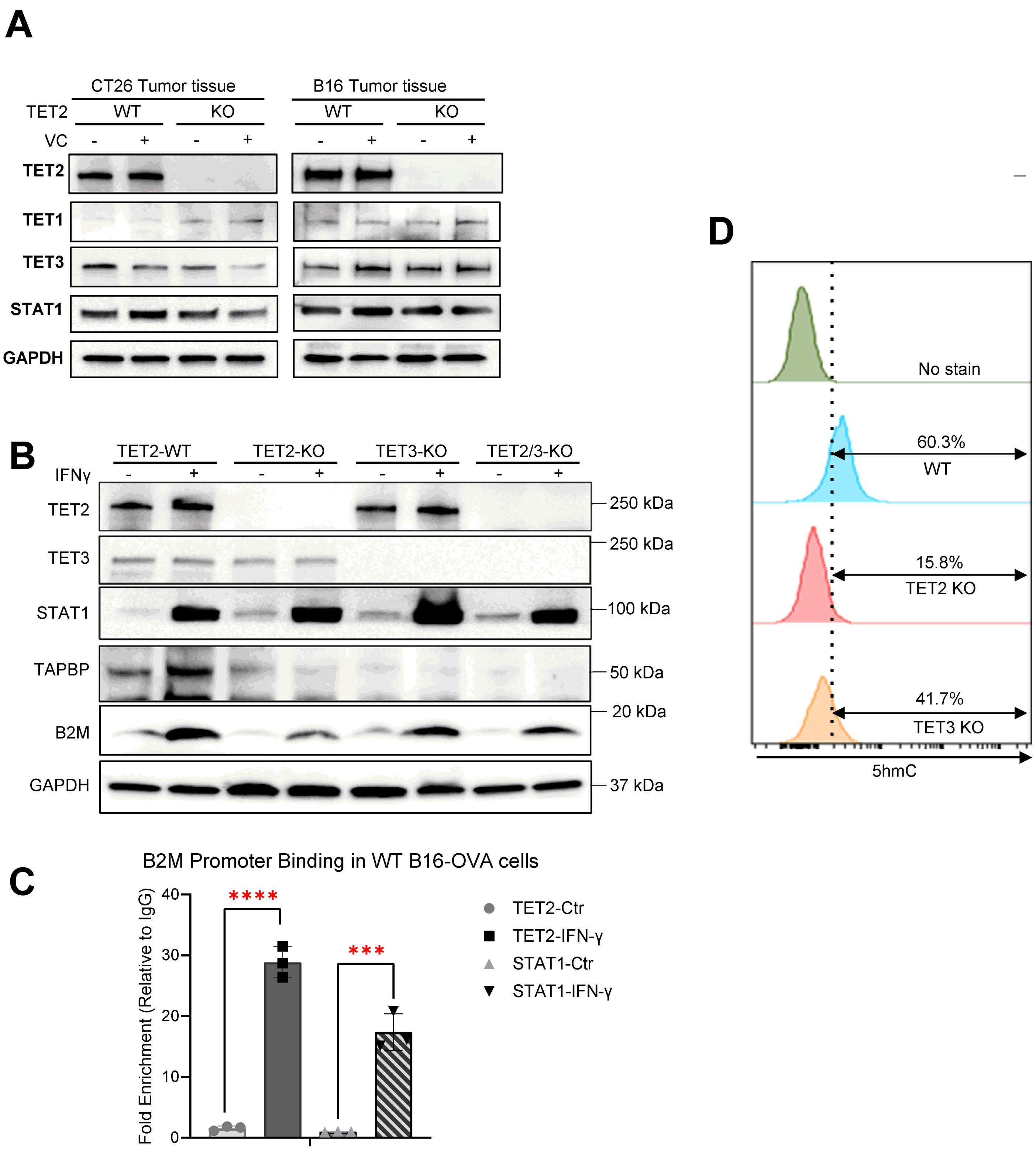

A
CT26 Tumor tissue
B16 Tumor tissue
TET2
WT
KO
VC
TET2
TET1
TET3
STAT1
GAPDH
B
TET2-WT
TET2-KO
TET3-KO
TET2/3-KO
IFNγ
TET2
TET3
STAT1
TAPBP
B2M
GAPDH
250 kDa
250 kDa
100 kDa
50 kDa
20 kDa
37 kDa
C
B2M Promoter Binding in WT B16-OVA cells
Fold Enrichment (Relative to IgG)
0
10
20
30
40
****
***
TET2-Ctr
TET2-IFN-γ
STAT1-Ctr
STAT1-IFN-γ
D
No stain
60.3%
WT
15.8%
TET2 KO
41.7%
TET3 KO
5hmC

Figure S1. The sequencing confirmation of TET2-KO CT-26 clones.
(A) WT or two TET2-KO CT-26 clones were generated using CRISPR-Cas9 gene editing technique and confirmed by DNA sequencing, the loss-of-function mutations in TET2-KO clones were aligned to WT sequences. (B) TET3 knockout and TET2/TET3 double knockout B16-OVA cells were generated by CRISPR-Cas9 gene editing technique and confirmed by DNA sequencing, the loss-of-function mutations in TET2-KO clones were aligned to WT sequences.

Figure S2. Characterization of Cluster 9. VC induced antigen presentation processes and molecular functions rely on the expression of TET2.
UMAP clustering for group 9 cells (see Fig. 2) (A) based on expression of established markers for dendritic cells and macrophages (B) (63). The top 10 most significantly enriched cellular processes in the WT tumor basal expression compared to TET2-KO in cluster C0 (C) or in cluster C3 (D) were presented based on GO analysis of the top 100 DEGs, and the antigen presentation processes were marked in red. The top 10 most significantly enriched molecular functions after VC stimulation in the WT B16-OVA tumor clusters C0 (E) or C2 (F) and in the TET2-KO tumor cluster C0 (G) or C2 (H) were determined by GO analysis of the top 100 DEGs for each cluster with antigen presenting functions marked in red.

Figure S3. VC induced MHC class I antigen presentation-related gene expression in the WT but not TET2-KO tumor cells.
The top 30 DEGs after VC treatment in WT tumor cluster C1 (A) or WT tumor cluster C2 (B) and TET2-KO tumor cluster C1 (C) or TET2-KO tumor cluster C2 (D) were annotated in the volcano plots according to their adjusted P values and genes involved in the MHC I antigen presentation were marked in red. (E) Representation of DEGs between WT tumor cluster 3 and TET2-KO cluster 3 annotated as volcano plots.

Figure S4. VC induced MHC class I antigen presentation related gene expression in the WT but not TET2-KO tumor cells.
The single-cell expression of MHC class I antigen presenting genes TAPBP (A), B2M (B), H2-D1 (C), H2-Aa (D) and immunoproteasome genes PSMB9 (E), PSMB10 (F) in the WT or TET2-KO B16-OVA tumor tissue treated with PBS control or VC were summarized. The relative expression of genes shown above was calculated by log2-normalized gene counts data. On the UMAP projection, purple indicates high level and yellow indicates low level with quantification presented in the violin plot above.

Figure S5. VC induced MHC class I antigen presentation related gene expression in the WT but not TET2-KO tumor cells.
(A) The expression of the three TET family proteins: TET1, TET2 and TET3 as well as STAT1 in WT or TET2-KO CT26 and B16-OVA tumor tissue as shown by western-blot. (B) Expression of TAPBP and B2M in TET2 WT and KO, TET3 KO, and TET2/3 KO B16-OVA cells following IFN- stimulation. (C) ChIP assay at the B2M promoter for TET2 and STAT1, following IFN- stimulation of B16 OVA cells. (D) 5hmC levels in WT or TET2-KO or TET3-KO B16-OVA cells as determined by FACS.

Figure 1A, Table

PBS	-				
Anti-PDL1	n.s.	-			
Anti-PDL1 + i.p. 1g/kg VitC	*	n.s.	-		
Anti-PDL1 + i.p. 4g/kg VitC	**	n.s.	n.s.	-	
Anti-PDL1 + i.v. 0.25g/kg VitC	***	n.s.	n.s.	n.s.	-
Anti-PDL1 + i.v. 1g/kg VitC	****	**	**	**	*
	PBS	Anti-PDL1	Anti-PDL1 + i.p. 1g/kg VitC	Anti-PDL1 + i.p. 4g/kg VitC	Anti-PDL1 + i.v. 0.25g/kg VitC

Figure 1B, Table

PBS	-				
Anti-PDL1	**	-			
Anti-PDL1 + i.p. 1g/kg VitC	**	n.s.	-		
Anti-PDL1 + i.p. 4g/kg VitC	****	n.s.	n.s.	-	
Anti-PDL1 + i.v. 0.25g/kg VitC	****	**	n.s.	n.s.	-
Anti-PDL1 + i.v. 1g/kg VitC	****	***	**	*	n.s.
	PBS	Anti-PDL1	Anti-PDL1 + i.p. 1g/kg VitC	Anti-PDL1 + i.p. 4g/kg VitC	Anti-PDL1 + i.v. 0.25g/kg VitC

	WT+PBS	WT+VC	WT+anti-PDL1	WT+anti-PDL1+VC	Tet2-KO+PBS	Tet2-KO+VC	Tet2-KO+anti-PDL1	Tet2-KO+anti-PDL1+VC
WT+PBS	-							
WT+VC	n.s.	-						
WT+anti-PDL1	****	****	-					
WT+anti-PDL1+VC	****	****	****	-				
Tet2-KO+PBS	n.s.	-	-	-	-			
Tet2-KO+VC	-	n.s.	-	-	n.s.	-		
Tet2-KO+anti-PDL1	-	-	****	-	n.s.	n.s.	-	
Tet2-KO+anti-PDL1+VC	-	-	****	****	n.s.	n.s.	n.s.	-

Figure 1D, Table

	WT+PBS	WT+VC	WT+anti-PDL1	WT+anti-PDL1+VC	Tet2-KO+PBS	Tet2-KO+VC	Tet2-KO+anti-PDL1	Tet2-KO+anti-PDL1+VC
WT+PBS	-							
WT+VC	n.s.	-						
WT+anti-PDL1	****	**	-					
WT+anti-PDL1+VC	****	****	n.s.	-				
Tet2-KO+PBS	n.s.	-	-	-	-			
Tet2-KO+VC	-	n.s.	-	-	n.s.	-		
Tet2-KO+anti-PDL1	-	-	**	-	n.s.	n.s.	-	
Tet2-KO+anti-PDL1+VC	-	-	-	****	n.s.	n.s.	n.s.	-

Figure 1E, Table
