# Supplementary material for: TET2 promotes tumor antigen presentation and T cell IFN-γ, which is enhanced by vitamin C
Source: JCI Insight. 2024 Nov 22;9(22):e175098. doi: 10.1172/jci.insight.175098 (PMC11601905; doi:10.1172/jci.insight.175098)
Supplement: Unedited blot and gel images [file jciinsight-9-175098-s079.pdf]

Figure 1. i.v injection of Vitamin C provides optimal, TET2-dependent anti-PD-L1 immunotherapy efficacy.

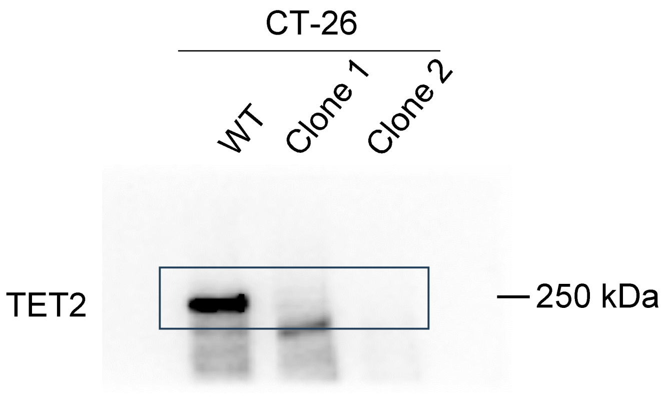

TET2-KO clones of CT-26 cells made by CRISPR/Cas9 system were confirmed by western blotting

Figure 1C

Figure 1. i.v injection of Vitamin C provides optimal, TET2-dependent anti-PD-L1 immunotherapy efficacy.

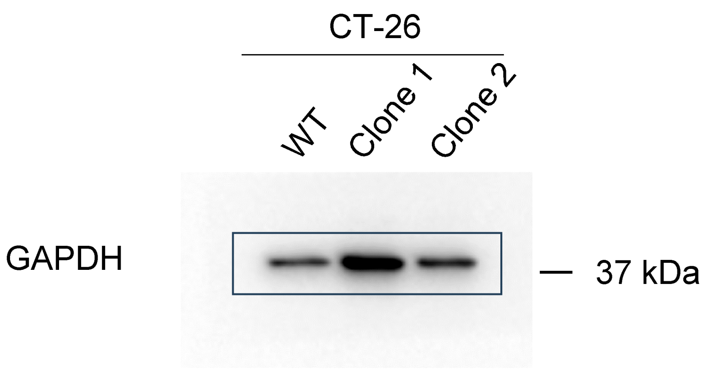

TET2-KO clones of CT-26 cells made by CRISPR/Cas9 system were confirmed by western blotting

Figure 1C

Figure 6. IFN-γ signal communication was identified in the WT tumor microenvironment and played a critical role in regulating MHC I gene transcription.

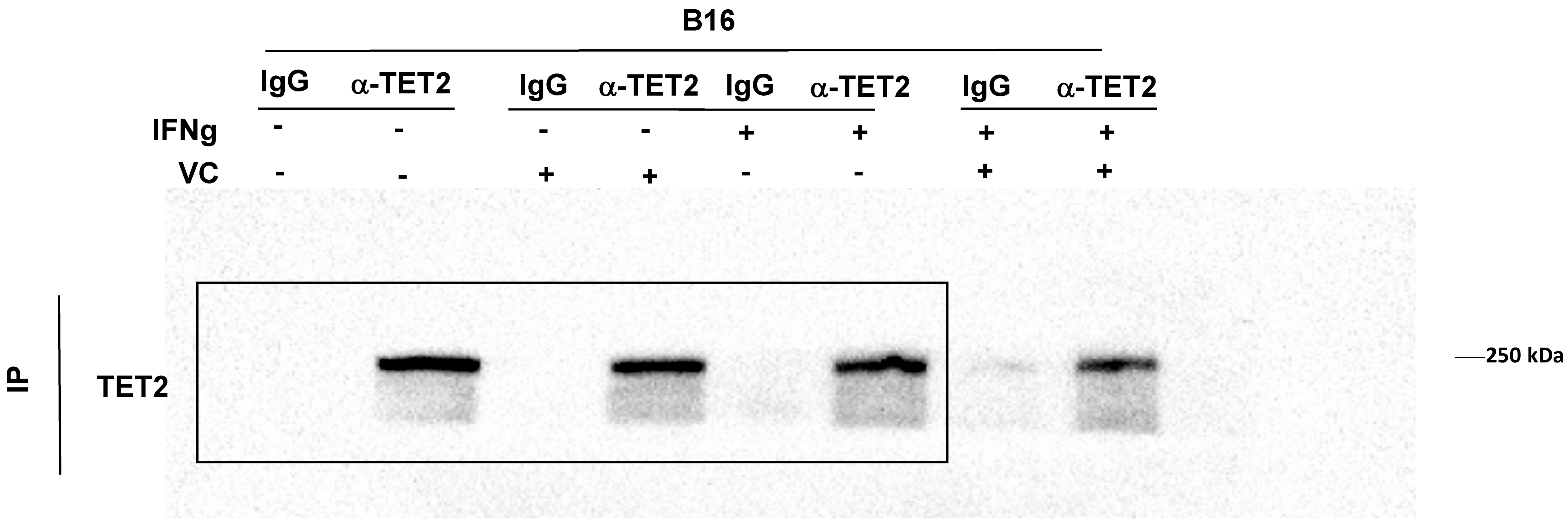

Protein Immunoprecipitation (IP) was conducted between TET2 and STAT1 in the presence of IFNg or VC treatment.

Figure 6D

Figure 6. IFN-γ signal communication was identified in the WT tumor microenvironment and played a critical role in regulating MHC I gene transcription.

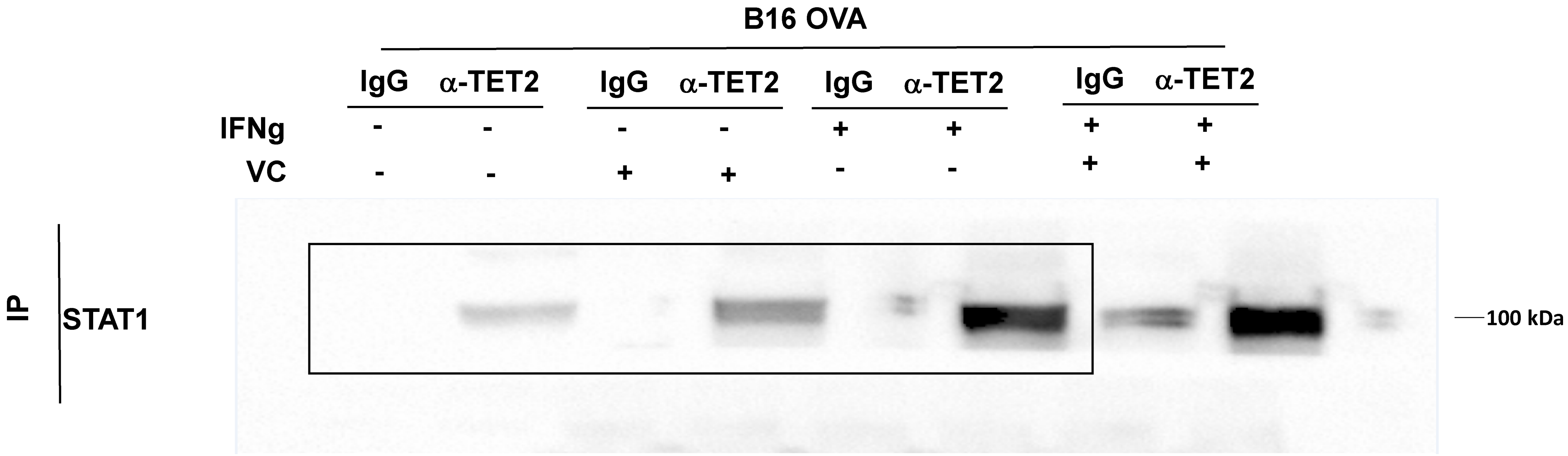

Protein Immunoprecipitation (IP) was conducted between TET2 and STAT1 in the presence of IFNg or VC treatment.

Figure 6D

Figure 6. IFN-γ signal communication was identified in the WT tumor microenvironment and played a critical role in regulating MHC I gene transcription.

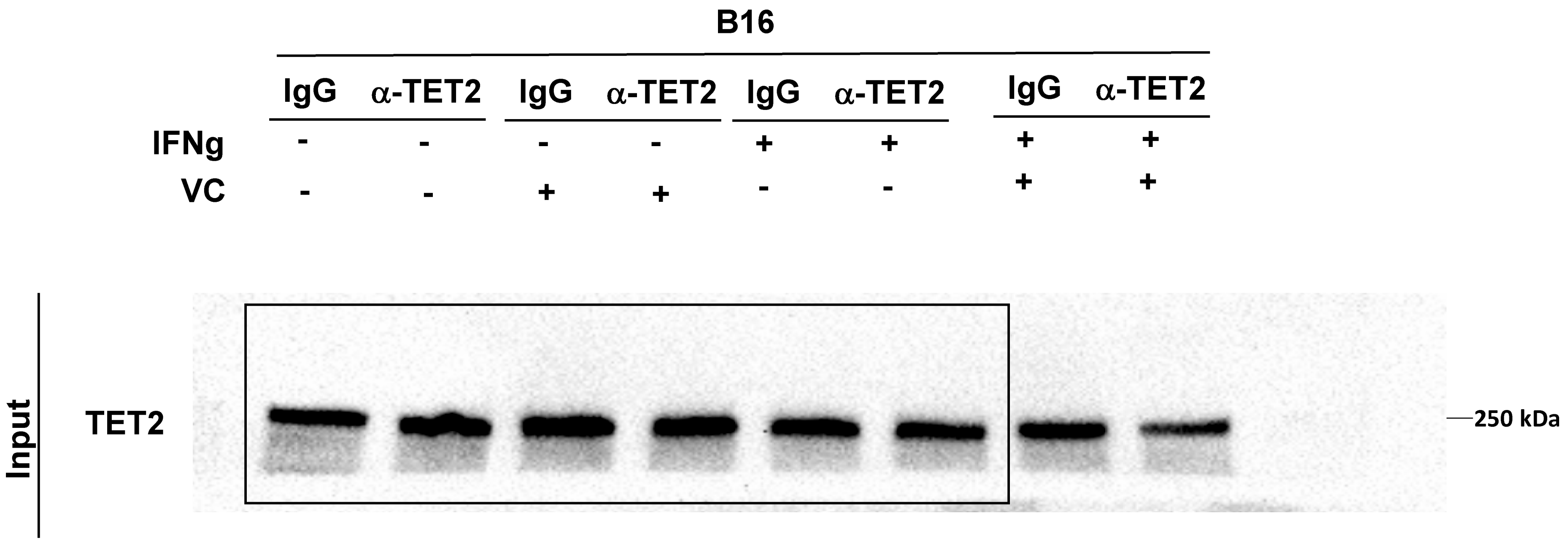

Protein Immunoprecipitation (IP) was conducted between TET2 and STAT1 in the presence of IFNg or VC treatment.

Figure 6D

Figure 6. IFN-γ signal communication was identified in the WT tumor microenvironment and played a critical role in regulating MHC I gene transcription.

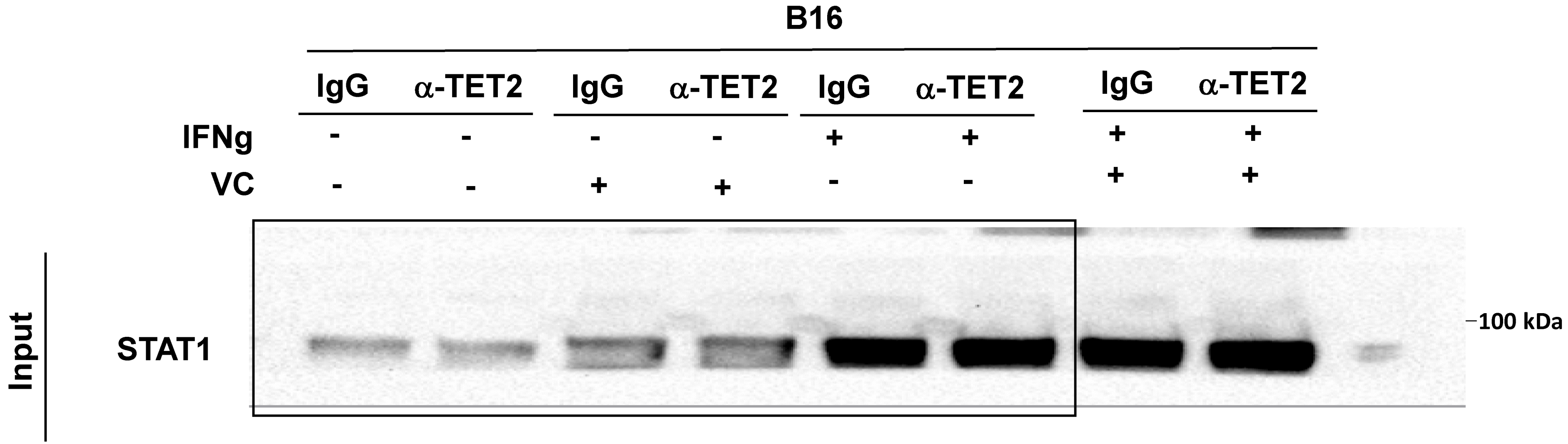

Protein Immunoprecipitation (IP) was conducted between TET2 and STAT1 in the presence of IFNg or VC treatment.

Figure 6D

Figure 6. IFN-γ signal communication was identified in the WT tumor microenvironment and played a critical role in regulating MHC I gene transcription.

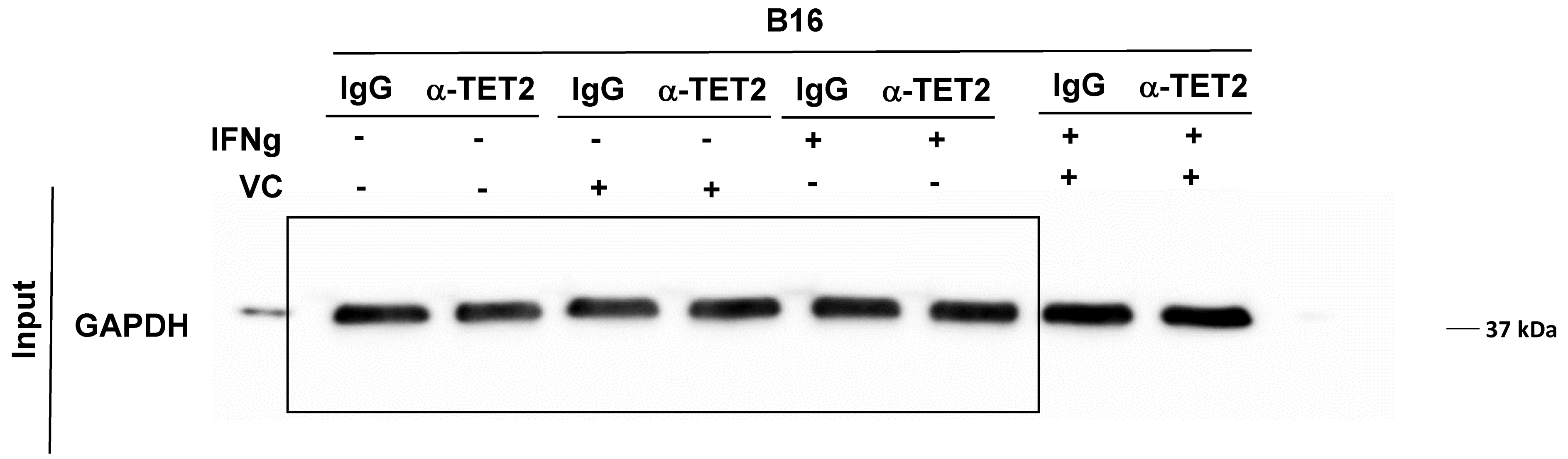

Protein Immunoprecipitation (IP) was conducted between TET2 and STAT1 in the presence of IFNg or VC treatment.

Figure 6D

Figure 6. IFN-γ signal communication was identified in the WT tumor microenvironment and played a critical role in regulating MHC I gene transcription.

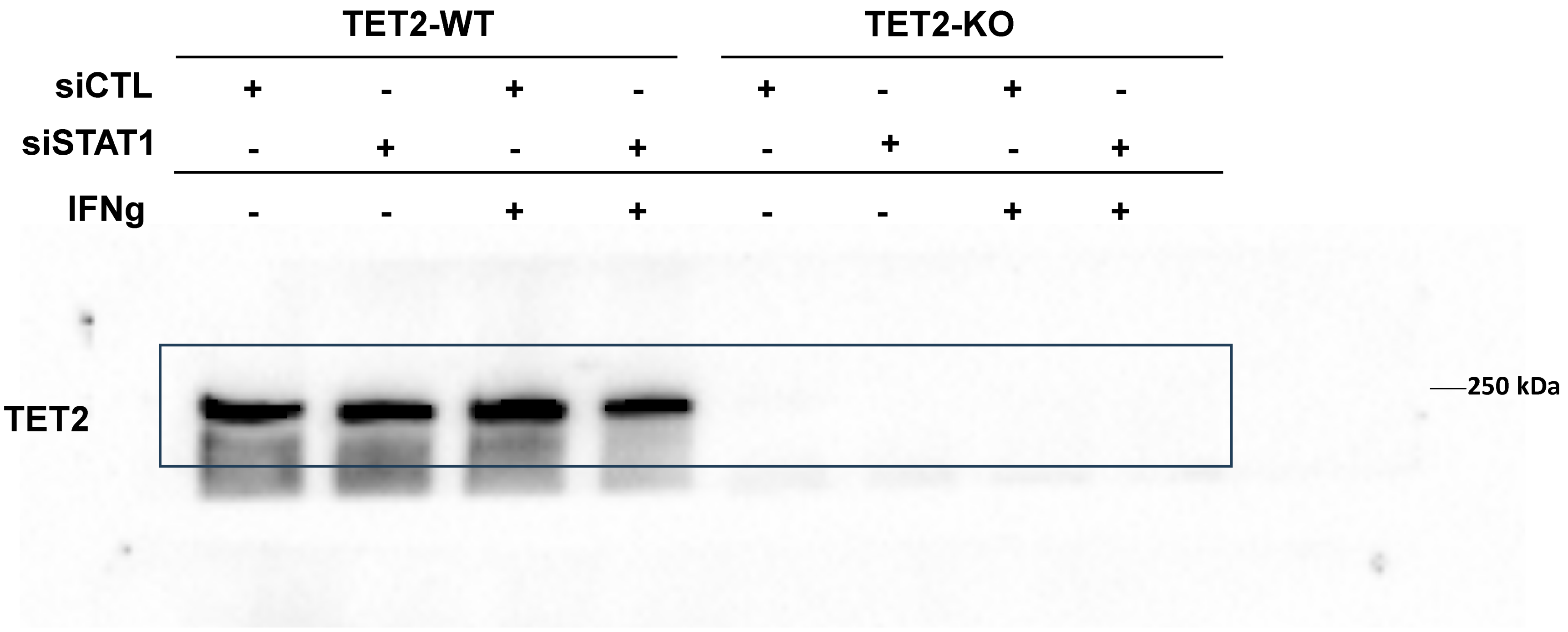

Protein expression of MHC I antigen-presenting genes TAP1, TAPBP was determined by Western-blot in the WT or TET2-KO B16-OVA cells under IFNg treatment or STAT1 siRNA knock down.

Figure 6F

Figure 6. IFN-γ signal communication was identified in the WT tumor microenvironment and played a critical role in regulating MHC I gene transcription.

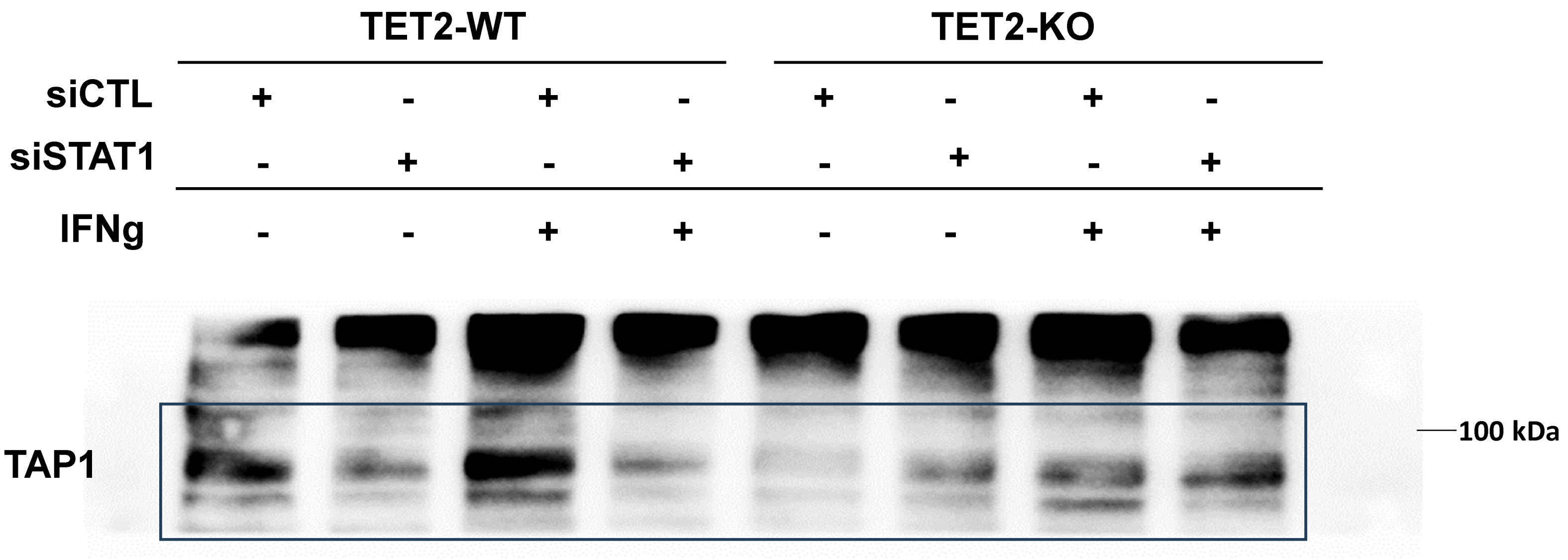

Protein expression of MHC I antigen-presenting genes TAP1, TAPBP was determined by Western-blot in the WT or TET2-KO B16-OVA cells under IFNg treatment or STAT1 siRNA knock down.

Figure 6F

Figure 6. IFN-γ signal communication was identified in the WT tumor microenvironment and played a critical role in regulating MHC I gene transcription.

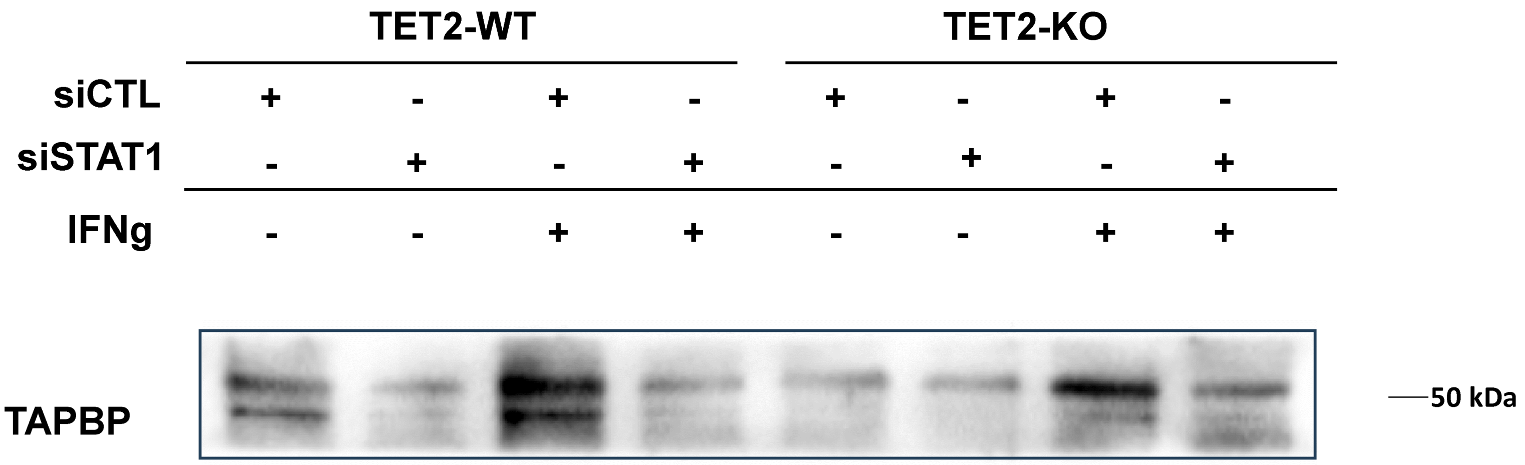

Protein expression of MHC I antigen-presenting genes TAP1, TAPBP was determined by Western-blot in the WT or TET2-KO B16-OVA cells under IFNg treatment or STAT1 siRNA knock down.

Figure 6. IFN-γ signal communication was identified in the WT tumor microenvironment and played a critical role in regulating MHC I gene transcription.

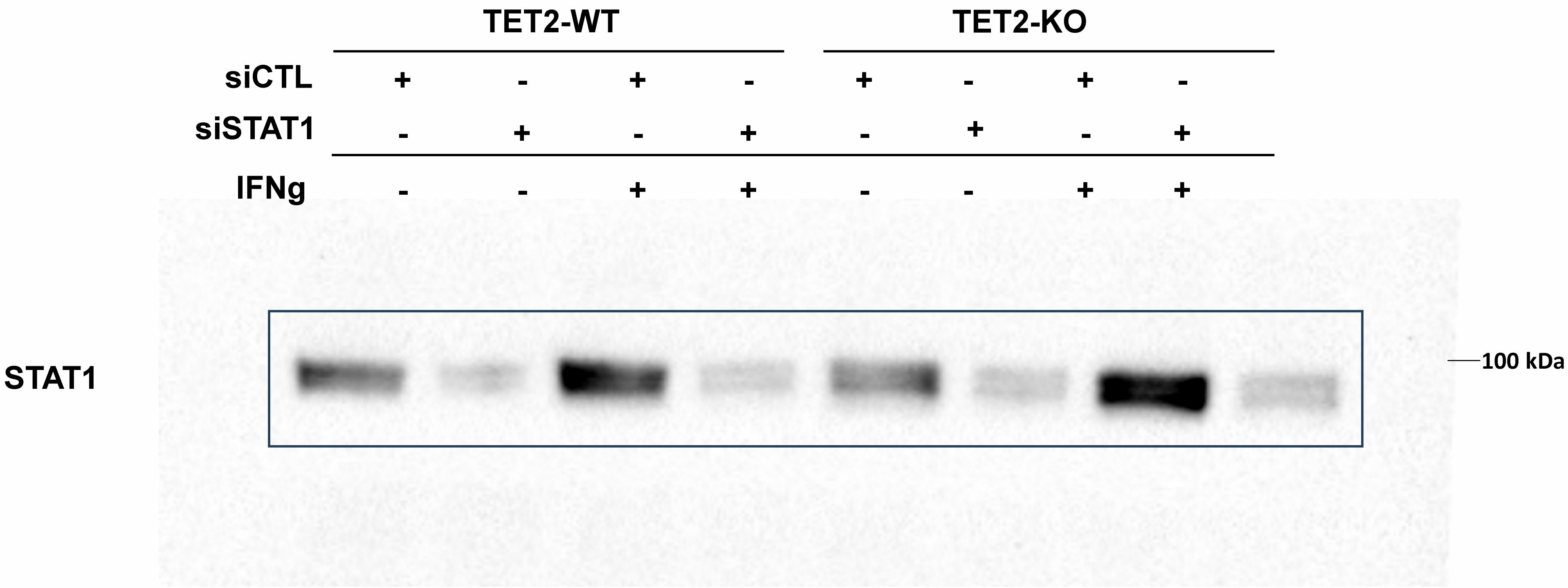

Protein expression of MHC I antigen-presenting genes TAP1, TAPBP was determined by Western-blot in the WT or TET2-KO B16-OVA cells under IFNg treatment or STAT1 siRNA knock down.

Figure 6F

Figure 6. IFN-γ signal communication was identified in the WT tumor microenvironment and played a critical role in regulating MHC I gene transcription.

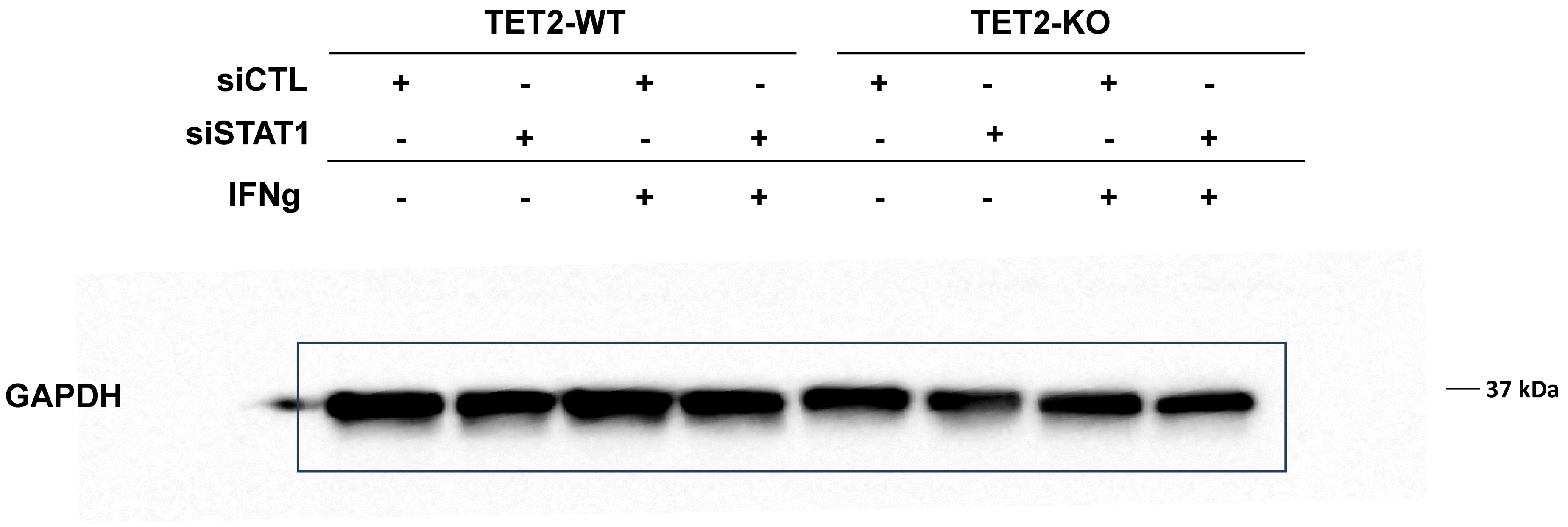

Protein expression of MHC I antigen-presenting genes TAP1, TAPBP was determined by Western-blot in the WT or TET2-KO B16-OVA cells under IFNg treatment or STAT1 siRNA knock down.

Figure 6F
